# ACBP/DBI neutralization for the experimental treatment of fatty liver disease

**DOI:** 10.1038/s41418-024-01410-6

**Published:** 2024-11-16

**Authors:** Omar Motiño, Flavia Lambertucci, Adrien Joseph, Sylvère Durand, Gerasimos Anagnostopoulos, Sijing Li, Vincent Carbonnier, Uxía Nogueira-Recalde, Léa Montégut, Hui Chen, Fanny Aprahamian, Nitharsshini Nirmalathasan, Maria Chiara Maiuri, Federico Pietrocola, Dominique Valla, Cédric Laouénan, Jean-François Gautier, Laurent Castera, QUID NASH Investigators, Dominique Valla, Cédric Laouénan, Jean-François Gautier, Laurent Castera, Anaïs Vallet-Pichard, Tiphaine Vidal-Trécan, Pauline Manchon, Valérie Paradis, Dominique Roulot, Christian Boitard, Benoit Terris, Hélène Bihan, Jean-Baptiste Julla, Thierry Poynard, Angélique Bzrustowski, Etienne Larger, Sébastien Czernichow, Stanislas Pol, Pierre Bedossa, Christophe Junot, Nathalie de Preville, Isabelle Durand Zaleski, Pierre-Emmanuel Rautou, Bernard Van Beers, Marco Dioguardi, Valérie Vilgrain, Jean-Marie Correas, Philippe Garteiser, Jean-Pierre Riveline, Mark Ibberson, Isabelle Martins, Guido Kroemer

**Affiliations:** 1 Centre de Recherche des Cordeliers, Inserm U1138, Université de Paris, Sorbonne Université, Equipe labellisée par la Ligue contre le cancer, Institut Universitaire de France, Paris, France; 2 Metabolomics and Cell Biology Platforms, Institut Gustave Roussy, Villejuif, France; 3 Unidad de Excelencia, Instituto de Biología y Genética Molecular (IBGM), Universidad de Valladolid - CSIC, Valladolid, Spain; 4 Grupo de Investigación en Reumatología (GIR), Instituto de Investigación Biomédica de A Coruña (INIBIC), Fundación Profesor Novoa Santos, A Coruña, Spain; 5 Department of Molecular Medicine and Medical Biotechnologies, University of Napoli Federico II, Naples, Italy; 6 Department of Bioscience and Nutrition, Karolinska Institute, Huddinge, Sweden; 7 Université Paris Cité, UMR1149 (CRI), Inserm, Paris, France; 8 Service hépatologie, AP-HP, Hôpital Beaujon, Clichy, France; 9 Université Paris Cité and Université Sorbonne Paris Nord, Inserm U1137, Laboratory “Infection, Antimicrobials, Modelling, Evolution” (IAME), Paris, France; 10 Département d’Epidémiologie Biostatistique et Recherche Clinique, AP-HP.Nord, Hôpital Bichat, Paris, France; 11 Institut Necker Enfants Malades, Inserm U1151, CNRS UMR 8253, IMMEDIAB Laboratory, Paris, France; 12 Centre Universitaire de Diabétologie et de ses Complications, AP-HP, Hôpital Lariboisiére, Paris, France; 13 Pôle de Biologie, Hôpital Européen Georges Pompidou, AP-HP, Institut Universitaire de France, Paris, France; 14 Service d’hépatologie, AP-HP, Groupe hospitalier Cochin, Paris, France; 15 Service d’anatomie et de cytologie pathologiques, AP-HP, Hôpital Beaujon, Clichy-la-Garenne, France; 16 Unité d’hépatologie, AP-HP, Hôpital Avicenne, Bobigny, France; 17 Université de Paris, Institut Cochin, U1016, Inserm, Paris, France; 18 Service de diabétologie, AP-HP, Groupe hospitalier Cochin, Paris, France; 19 Service d’anatomie et de cytologie pathologiques, AP-HP, Groupe hospitalier Cochin, Paris, France; 20 France Université Paris 13, EA 3412, Bobigny, France; 21 Service Endocrinologie, Diabéte, Nutrition, AP-HP, Hôpital Avicenne, Bobigny, France; 22 BioPredictive, Paris, France; 23 INSERM Délégation régionale Paris 5 / Paris 7, Paris, France; 24 Université de Paris, UMR1153 (METHODS Team, CRESS), Inserm, Paris, France; 25 Service de nutrition, centre spécialisé Obésité, APHP, Hôpital Européen Georges Pompidou, Paris, France; 26 Liverpat, Paris, France; 27 Commissariat à l’Energie Atomique (CEA), Paris, France; 28 Servier, Suresnes, France; 29 Unité de Recherche Clinique en Économie de la Santé, Île-de-France, Université de Paris, AP-HP, Paris, France; 30 Service de Radiologie, AP-HP, Hôpital Beaujon, Clichy, France; 31 Sorbonne Université, CNRS, INSERM Laboratoire d’Imagerie Biomédicale, Paris, France; 32 Service d’Imagerie Adulte, AP-HP, Hôpital Necker Enfants Malades, Paris, France; 33 Vital-IT Group, Swiss Institute of Bioinformatics, Lausanne, Switzerland

**Keywords:** Metabolic disorders, Macroautophagy

## Abstract

Acyl-CoA binding protein (ACBP), also known as diazepam-binding inhibitor (DBI), is an extracellular checkpoint of autophagy. Here, we report that patients with histologically confirmed metabolic-associated steatohepatitis (MASH) or liver fibrosis exhibit elevated levels of circulating ACBP/DBI protein as compared to non-affected controls. Plasma ACBP/DBI strongly correlated with the NAFLD and FIB4 scores in patients, and these correlations were independent of age and body mass index. We studied the capacity of a monoclonal antibody (mAb) neutralizing mouse ACBP/DBI to combat active liver disease in several mouse models, in which steatohepatitis had been induced by four different protocols, namely, (i) methionine/choline-deficient diet, (ii) Western style diet (WD) alone, (iii) WD combined with the hepatotoxic agent CCl_4_, and (iv) a combination of CCl_4_ injections and oral ethanol challenge. Injections of anti-ACBP/DBI mAb attenuated histological, enzymological, metabolomic and transcriptomic signs of liver damage in these four models, hence halting or reducing the progression of non-alcoholic and alcoholic liver disease. Steatosis, inflammation, ballooning and fibrosis responded to ACBP/DBI inhibition at the preclinical level. Altogether, these findings support a causal role of ACBP/DBI in MASH and liver fibrosis, as well as the possibility to therapeutically target ACBP/DBI.

## Introduction

Acyl coenzyme A (CoA) binding protein (ACBP), which is encoded by *diazepam binding inhibitor* (*DBI*), is a precursor of neuropeptides that act within the hypothalamus [1, 2]. Outside of the central nervous system, ACBP/DBI, which is close-to-ubiquitously expressed, has a dual function [3]. As an intracellular protein, ACBP/DBI binds to various lipid species including medium-chain fatty acid-CoA esters and stimulates fatty acid metabolism in a variety of cell types [4, 5]. As an extracellular protein, ACBP/DBI interacts with γ-aminobutyric acid (GABA) receptors of the A type (GABA_A_R), specifically to the γ2 subunit (GABRG2), which also binds the benzodiazepine diazepam, to act as a positive allosteric modulator [6, 7]. Through this ligand-receptor interaction, ACBP/DBI modulates chloride influx into target cells, thereby mediating its physiological effects that include the inhibition of autophagy and lipophagy, as well as the stimulation of lipo-anabolic reactions [8]. Thus, addition of ACBP/DBI antagonists to primary hepatocytes induces autophagic flux [9]. Similarly, ACBP/DBI neutralization by means of a monoclonal antibody (mAb) stimulates autophagic flux measured in the liver in the absence or presence of leupeptin injections [9, 10]. At the whole-body level, recombinant ACBP/DBI protein injected into peripheral locations enhances food intake and weight gain [8, 11]. Although ACBP/DBI does not cross the blood–brain barrier, it inhibits anorexigenic neurons in the hypothalamus, thereby stimulating appetite [12].

There are several strategies to inhibit the ACBP/DBI-GABRG2 interaction, namely (i) the induction of autoantibodies against ACBP/DBI by active immunization [13], (ii) passive immunization against ACBP/DBI by injection of neutralizing antibodies [8], (iii) inducible knockout of the *Dbi* gene [8], or (iv) point mutation F77I of GABRG2 to abolish its interaction with ACBP/DBI without affecting that with GABA [7, 9]. All these manipulations have similar consequences on organismal physiology, namely, the induction of autophagy in various organs (e.g., heart, liver, and lung), the autophagy-dependent protection of such organs against physical and chemical damage [8, 10, 14], as well as a reduction in food intake, especially in the context of high-fat diet (HFD) and leptin deficiency [8, 11]. Obviously, the only potentially translatable mode of ACBP/DBI inhibition consists in the administration of neutralizing monoclonal antibodies.

With respect to liver disease, we reported that ACBP/DBI neutralization protected against acute hepatic damage by two chemical agents (acetaminophen/paracetamol, as well as the T-cell activating lectin concanavalin A) and two physical insults (ischemia/reperfusion damage inflicted by temporary occlusion of the portal triade, as well as cholestasis induced by bile duct ligation). Moreover, in the prophylactic setting, anti-ACBP/DBI mAb prevented MASH induced by high-fat (HFD) or methionine/choline-deficient diet (MCD), as well as fibrosis induced by CCl_4_ [10, 15]. These favorable effects could be explained by the induction of autophagy, the protection of hepatocytes (which express GABRG2) [9] against unwarranted cell death, as well as by anti-inflammatory and antioxidant effects [10].

Encouraged by these preclinical findings, we investigate whether ACBP/DBI might be associated with the most frequent human hepatic pathology, which is metabolic-associated fatty liver disease (MAFLD) [10, 16]. Of note, the notion of MAFLD amalgamates two previously opposed diseases, namely non-alcoholic fatty liver disease (NAFLD) and alcoholic liver disease (AFLD). This reflects a paradigm shift caused by the observation that NAFLD is mediated by alcohol (ethanol) produced by microbial (bacterial or fungal) fermentation of carbohydrates in the gut [17]. At the preclinical level, we explored the possibility that ACBP/DBI neutralization might be used for therapeutic interventions on active liver disease rather than for prophylaxis of liver damage, as we previously reported [10, 15]. Our results plead in favor of the implication of ACBP/DBI in human MAFLD and suggest the possibility of therapeutic interventions based on ACBP/DBI neutralization.

## Material and methods

### Quantitative imaging in Diabetes—NASH (QUID-NASH) cohort

This study (ClinicalTrials.gov identifier NCT03634098, https://rhu-quidnash.com/) [18–21] enrolled patients with type 2 diabetes with metabolic dysfunction-associated steatotic liver disease (MASLD) who underwent a liver biopsy as part of standard care for the evaluation of unexplained liver alterations and a suspicion of metabolic dysfunction associated steatohepatitis (MASH). As reported [22] inclusion criteria for MASLD patients were based on the absence of alcohol intake (<20 g per day); the absence of causes for chronic liver disease except MASLD; a persistent increase in transaminase levels above 20 IU/L in women and 30 IU/L in men; absence of evidence for another cause for liver disease at liver biopsy (e.g. granulomas). Liver biopsy interpretation by a central expert pathologist was as reported [22]. Patients with a positive fibrosis score (EPoS 1-8) were considered as positive for this parameter. Non-invasive scores for diagnosis of fibrotic MASH were calculated as reported [21]. We further studied patients from whom a liver biopsy was taken during programmed laparoscopic cholecystectomy and who were diagnosed with histologically normal livers (non-MASLD patients). All non-MASLD patients included in this study had normal fasting glucose, cholesterol and triglycerides, normal serum ALT levels, and negative anti-HBV, anti-HCV and anti-HIV tests. In addition, all of them drank <20 g of alcohol per day and none did use potentially hepatotoxic drugs. The study was performed in agreement with the Declaration of Helsinki, in strict compliance with local and national laws. Written informed consent was obtained from all patients before inclusion in the study, and the Institution’s Human Ethics Committee approved the study procedures. Continuous data were described as mean and standard deviation and categorical data are presented as numbers and percentages of total. Comparison of characteristics between patients with and without MASH was performed using Student’s *t*-test or Wilcoxon test, as appropriate. Normality of data was assessed using Shapiro–Wilk normality test. Correlation between metabolic and anthropometric parameters were assessed using Pearson correlation coefficient and adjustment were made using multivariate logistic regression models. *P* values less than 0.05 were considered statistically significant, and all statistical tests were 2-sided. Statistical analyses were performed using R version 3.4.2 (R Foundation for Statistical Computing, Vienna, Austria; https://www.R-project.org/).

### Chemicals and reagents

Reagents were obtained from Qiagen (Hilden, Germany), Millipore (MA, USA), Randox (Antrim, UK), Roche Applied Science (Upper Bavaria, Germany) and Sigma Aldrich (MO, USA). Reagents for electrophoresis were obtained from Thermo Fisher Scientific (MA, USA) and BioRad (CA, USA). Antibodies were from Abcam (TX, USA), Abnova (Taipei, Taiwan), Cell Signaling (MA, USA) and Sigma Aldrich.

### Biochemical assays

Plasma ALT (alanine transaminase) and AST (aspartate transaminase) activity was determined by colorimetric kits (Randox, UK) accordingly with the manufacturer’s instructions.

### Animal experimentation

Wild-type (Wt) C57BL/6 mice (Envigo, Gannat, France) were bred and maintained according to the FELASA guidelines and local guidelines from the Animal Experimental Ethics Committee Charles Darwin (CEEA-005; protocols #25000-2020040718432518, #31411-2021050411267667 v3, #34537-2022010210461547, #34538-2022010215486066, and #34589-2022010916126141). All experimental protocols were reviewed and approved by the CEEA-005 Ethics Committee. Mice were housed in a temperature-controlled environment with 12 h light/dark cycles and were provided with chow and water ad libitum. All mice were sacrificed, plasma were collected and livers were snap-frozen in liquid nitrogen and stored at −80 °C, or fixed in 4% buffered paraformaldehyde overnight at 4 °C and embedded in paraffin.

### Neutralization of ACBP/DBI by passive immunization

The custom-made monoclonal antibody against DBI (anti-DBI, clone 7G4a, Fred Hutch Cancer Center) [8] or isotype IgG (Bioxcell, NH, USA) was administered in vivo (standard dose: 5 µg/g body weight, B.W., intraperitoneally, i.p., in 200 µL) at the indicated periodicity. This protocol of periodic anti-DBI neutralization reduces circulating ACBP/DBI plasma level to approximately 50% of the level observed in mice receiving the control isotype IgG [8, 10].

### In vivo MASH models

Male 8 weeks old mice were fed with three major types of diets to induce MASH (i) Methionine and choline deficient diet (MCD) (ii) Western diet (WD) or (iii) WD plus carbon tetrachloride (CCl_4_) [23]. The animals were randomized to start the neutralization of ACBP/DBI by passive immunization while maintaining the diets in a curative setting. (i) Mice were fed with regular chow diet (RCD) or MCD (AIN-76 safe diet, Essingen, Germany), and after 4 weeks, two groups of mice were injected with anti-DBI or IgG by several doses for 2 weeks. (ii) Mice received a Western diet (WD, i.e., a high-fat, high-fructose and high-cholesterol diet, MD.120528, Envigo), along with high-sugar water (23.1 g/L fructose plus 18.9 g/L glucose), and after 16 weeks, anti-DBI or IgG was regularly administrated for 4 weeks. (iii) Mice were fed with WD high-sugar water and then received weekly i.p. injections of CCl_4_ (1:10 diluted in corn oil, Sigma-Aldrich) at a final dose of 2 μL/g of body weight [24], and after 10 weeks, groups of mice were injected with repeated doses of anti-DBI or IgG for 4 weeks. Control animals were injected i.p. with the vehicle olive oil (Sigma Aldrich). In all experiments, fresh food was provided at least once per week and body weights were recorded weekly.

### In vivo ASH model

To induce model of advanced alcoholic steatohepatitis (ASH) with fibrosis, male 8 weeks old mice were fed with ethanol liquid diet in combination with CCl_4_ injections as described [25]. Briefly, mice were acclimated with Lieber-DeCarli liquid diet [26]. After 3 days, mice were gradually fed with Lieber-DeCarli liquid diet plus 2% ethanol (vol/vol) for a period of 2 weeks, which was increased to 4% for 2 weeks and then 5% for 4 weeks. During the treatment with ethanol, 0.5 µl/kg CCl_4_ or olive oil was administrated i.p. biweekly over 8 weeks. One day before the end of week 4, some mice were injected with IgG or anti-DBI (5 µg/g) and injected biweekly with the same antibodies until week 8. Mice were euthanized 24 h following the last dose of CCl_4_.

### Histopathology

Liver sections, sliced to 5 µm thickness from paraffin blocks, were stained with hematoxylin-eosin-safranin (HES) or Sirius Red and were evaluated by an experienced pathologist blinded to the features of the animal groups. All slides were scanned with an AxioScan Z1 microscope (Carl Zeiss, Jena, Germany). The NAFLD activity score was assessed using the NAFLD scoring system for mouse models [27]. Briefly, steatosis grade was classified as follows: grade 0, 1, 2, and 3 with macrovesicular steatosis present in less than 5%, 5% to 33%, 34% to 66%, and more than 66% of the hepatocytes, respectively. Lobular inflammation was scored as follows: 0 (no foci), 1 (<2 foci), 2 (2-4 foci), and 3 (>4 foci). Ballooning was classified as 0 (none), 1 (few balloon cells), and 2 (many balloon cells). The NAFLD activity score was calculated for each liver biopsy based on the sum of scores for steatosis, inflammation, and ballooning. In addition, liver fibrosis staging (Metavir score) was defined as follows: 0 (none), 1 (perisinusoidal and/or pericentral), 2 (incomplete central/central bridging fibrosis), 3 (complete central/central bridging fibrosis), and 4 (definite cirrhosis). The severity of hepatic ASH was graded according to Suzuki’s criteria on a scale from 0 to 4. None as 0%, minimal as 10%, mild as 11–30%, moderate as 30-60%, and severe as >60% necrosis, congestion, or centro-lobular ballooning were assigned as grade 0, 1, 2, 3, and 4, respectively [28].

### Liver extracts

Tissues were homogenized in 2 cycles for 20 s at 5500 rpm using a Precellys 24 tissue homogenator (Bertin Technologies, Montigny-le-Bretonneux, France) in 20 mM Tris buffer (pH 7.4) containing 150 mM NaCl, 1% Triton X-100, 10 mM EDTA and Complete® protease inhibitor cocktail (Roche Applied Science) for protein or in QIAzol (Qiagen) for RNA. Protein homogenates were centrifuged at 12,000 × *g* at 4 °C for 15 min and supernatants were collected. Protein concentrations in supernatants were analyzed by the bicinchoninic acid technique (BCA protein assay kit, Thermo Fisher Scientific). Homogenate RNA was purified with RNeasy Mini Kit (Qiagen) according to the manufacturer’s instructions. The concentration and integrity of total RNA were analyzed using electrophoretic separation on microfabricated chips in an Agilent 2100 Bioanalyzer System (Agilent, CA, USA).

### Whole transcriptome analysis

For RNA-sequencing library preparation, 1.5 μg total RNA per sample was analyzed by means of on NovaSeq 6000 PE150 instrument (2 × 150 bp, 40 million reads per sample). For RNA-sequencing data analysis, pseudo-alignment and quantification were performed using the HISAT2 algorithm (reference genome GRCm39). Subsequently, correlation analysis of principal component study and differential expression analysis were performed with the DESeq2 package. The analyses of the differential gene expression were ran using the parametric Wald test with Benjamini–Hochberg adjustment (p-adj). Genes were expressed using Z-score normalization and were considered differentially expressed log2 (fold change) (cut-off) was ≥1.5 or ≤1.5 with adjusted *p* values < 0.05 and and then subjected to GSEA (Gene Set Enrichment Analysis), based on gene ontology (GO) and Kyoto Encyclopedia of Genes and Genomes (KEGG). R software was used to draw the graphs.

### Immunodetection of proteins

For western blot analyses, protein extracts were boiled for 5 min in Laemmli sample buffer, and equal amounts of protein (20–30 μg) were separated on 4–12% Bis-Tris acrylamide precast gels (Thermo Fisher Scientific) and electro-transferred to PDVF membranes (BioRad). The transfer of proteins from PDVF membranes was conducted at a constant voltage of 100 V at 4 °C for 1 h30 min. Unspecific binding sites of the membranes were saturated by incubating for 1 h in 0.05% Tween 20 (v:v in TBS) supplemented with 5% non-fat powdered milk (w:v in TBS). Subsequently, proteins were determined by overnight incubation of membranes with primary antibodies specific for α-SMA (#A2547, Sigma Aldrich), collagen 1A1 (#234167, Sigma Aldrich), MAP1LC3B/LC3B (#2775, Cell signaling), and SQSTM1/p62 (#H00008878-M01, Abnova). Glyceraldehyde-3- phosphate dehydrogenase antibody (#2118, Cell signaling) was used to control equal loading of lanes. The blots were revealed using appropriate horseradish peroxidase (HRP)-labeled secondary antibodies (Southern Biotech, AL, USA) plus SuperSignal West Pico chemoluminescent substrate (Thermo Fisher Scientific). Different exposure times were utilized for each blot with a charged coupling device camera in a luminescent image analyzer LAS 4000 (GE Healthcare, IL, USA) to ensure the linearity of the band intensities. Quantification of proteins was carried out by densitometric analysis of the bands using ImageJ software (http://imagej.nih.gov) and was expressed as relative expression levels.

### Human ACBP/DBI ELISA

High binding 96 well plates (Corning) were coated with antibody using the following procedure: 100 µL/well of anti-ACBP/DBI capture antibody (Human: MBS768488), diluted in PBS at a final concentration of 1 μg/mL, was added and incubated for overnight at 4 °C. The plate was rinsed twice with washing buffer (0.05% Tween 20 in PBS) before the addition of 100 µL sterile blocking buffer (1% BSA, 0.05% Tween 20 in PBS) to each well and after 2 h of incubation at room temperature. One hundred microliters of samples or standard, i.e., recombinant human ACBP/DBI protein generated as reported [29], were added (human serum 1/50 dilution). Plates were incubated for 2 h at RT and then rinsed 3 times with washing buffer before the addition of 100 µL of anti-ACBP/DBI detection antibody (Human: LS-C299614), diluted to 1 μg/mL in PBS. Plates were incubated for 1 h at RT, rinsed 3 times with washing buffer, and incubated for 30 min at RT with 100 µL of HRP Avidin diluted in PBS (1/5000 for human). Plates were washed 4 times with washing buffer, 100 µL of 1-Step Ultra TMB-ELISA substrate solution (Thermo Fisher Scientific, #34029) was added and incubated for 10–30 min at RT in the dark until colorizing. Immediately after 50 µL of stop solution (2 M H_2_SO_4_) was added and the absorbance was measured at 450 nm in a microplate reader (FLUOstar OPTIMA).

### Liver and plasma preparation and metabolomic analysis

Livers (30 mg) were transferred to 2 mL-homogenizer tubes with ceramic beads (Hard Tissue Homogenizing CK28, 2.8 mm zirconium oxide beads; Precellys, Bertin Technologies), containing 1 mL of ice-cold extraction mixture (metOH/water, 9/1, −20 °C, with a cocktail of internal standards). The samples were homogenized in Precellys 24 tissue homogenize (3 cycles of 20 s/5000 rpm), followed by centrifugation (10 min at 15,000 × *g*, 4 °C), and collection of the supernatants. For plasma metabolomics, samples (25 μL) were mixed with 250 µL of the of ice-cold extraction mixture, allowing protein precipitation and metabolite extraction, then vortexed and centrifuged (10 min at 15,000 × *g*, 4 °C). After liver homogenate or plasma centrifugation, supernatants were collected and divided in several fractions, and treated following published protocols [10].

Analyses of bile acids, polyamines, and small/polar organic acids analyses were performed by LC–MS/MS with a 1290 UHPLC (Ultra-High Performance Liquid Chromatography) coupled to a QQQ 6470 (Agilent Technologies). Bile acids were subjected to multiple reaction monitoring (MRM) in negative polarity, when gas temperature was set to 310 °C with a gas flow of 9 L/min. Capillary voltage was set to 4.5 kV. Ten microliters of sample were injected on a Poroshell 120 EC-C8 1200bars (P/N 981758-902, 100 mm × 2.1 mm particle size 1.9 µm) column (Agilent technologies), protected by an XDB-C18 (5 mm × 2.1 mm particle size 1.8 μm) guard column and heated at 40°C in a Pelletier oven. The gradient mobile phase consisted of water with 0.2% of formic acid (A) and acetonitrile/isopropanol (1/1; v/v) (B). The flow rate was set to 0.5 mL/min, and the gradient was generated as follows: initial condition was 70% phase A and 30% phase B, changing to 38% phase B over 2 min, kept at 28% phase B for 2 minutes, and finally switching from 38% to 60% phase B over half a minute. Columns were washed using 98% mobile phase B for 2 min and equilibrated using 30% mobile phase B for 1.5 min before repetition of the cycle with another sample. The autosampler was kept at 4°C [30]. Polyamines were analyzed by MRM analysis in positive polarity, at a gas temperature of 350°C with a gas flow of 12 l/min. The capillary voltage was set to 2.5 kV. Ten μl of sample were injected on a Kinetex C18 (150 mm × 2.1 mm particle size 2.6 µm) column from Phenomenex, protected by a C18 (5 mm × 2.1 mm) guard column and heated at 40°C in a Pelletier oven. The gradient mobile phase consisted of water with 0.1% of heptafluorobutyric acid (HFBA, Sigma-Aldrich) (A) and acetonitrile with 0.1% of HFBA (B). The flow rate was set to 0.4 ml/min, and the gradient was generated as follows: the initial condition was 95% phase A and 5% phase B. Molecules were then eluted using a gradient from 5% to 30% phase B over 7 min. The column was washed using 90% mobile phase B for 2.25 min and equilibrated using 5% mobile phase B for 4 min [30]. Small organic acid ketone bodies and polar metabolites (so-called SF method) were analyzed by MRM in both positive and negative polarity, and gas temperature was set to 300°C with a gas flow of 12 L/min. Capillary voltage was set to 4 kV in the positive and 5 kV in the negative polarity mode. Ten μl of sample were injected into a Zorbax Eclipse XDB- (P/N 981758-902, 100 mm × 2.1 mm particle size 1.8 µm 1200 bars) column from Agilent technologies, protected by a XDB-C18 (5 mm × 2.1 mm particle size 1.8 μm) guard column and heated at 50°C in a Pelletier oven. Gradient mobile phase consisted of water with 0.1% of formic acid (A) and 0.1% of formic acid in acetonitrile (B). Flow rate was set to 0.7 mL/min, and the gradient was generated as follows: the initial condition was 80% phase A and 20% phase B for 3 minutes, changing to 45% phase B over 4 minutes before rinsing and equilibrating before next injection. Widely targeted GC-MS/MS and pseudo-targeted analysis by UHPLC-HRAM (ultra-high performance liquid chromatography—high-resolution accurate mass) was performed on a U3000 (Dionex)/Orbitrap coupled to a q-Exactive (Thermo). All targeted treated data were merged and cleaned with a dedicated R (version 4.0) package (@Github/Kroemerlab/GRMeta).

### Data analyses

Data are represented as means ± SEM. Graphpad was used for identifying outliers. Unpaired two-tailed Student’s t-test or one-way analysis of variance (ANOVA) followed by Bonferroni post hoc multiple comparison test were used for dual or multiple comparisons, respectively. The Wilcoxon test was used for analyzing body weight curves**’**. Analyses were performed using the statistical software GraphPad Prism 9. For RNAseq analysis, p value from multiple groups was calculated by Kruskal Wallis test followed by Dunn´s post hoc test. For metabolic analysis, the Mann-Whitney test and Kruskal Wallis test followed by Dunn’s post hoc test were used to calculated p values for dual or multiple groups, respectively. All targeted treated data were merged and cleaned with a dedicated R (version 3.4) package (@Github/Kroemerlab/GRMeta). A *p* < 0.05 was considered as statistically significant.

## Results

### Elevated plasma ACBP/DBI levels in patients with MASLD

**Fig. 1:**
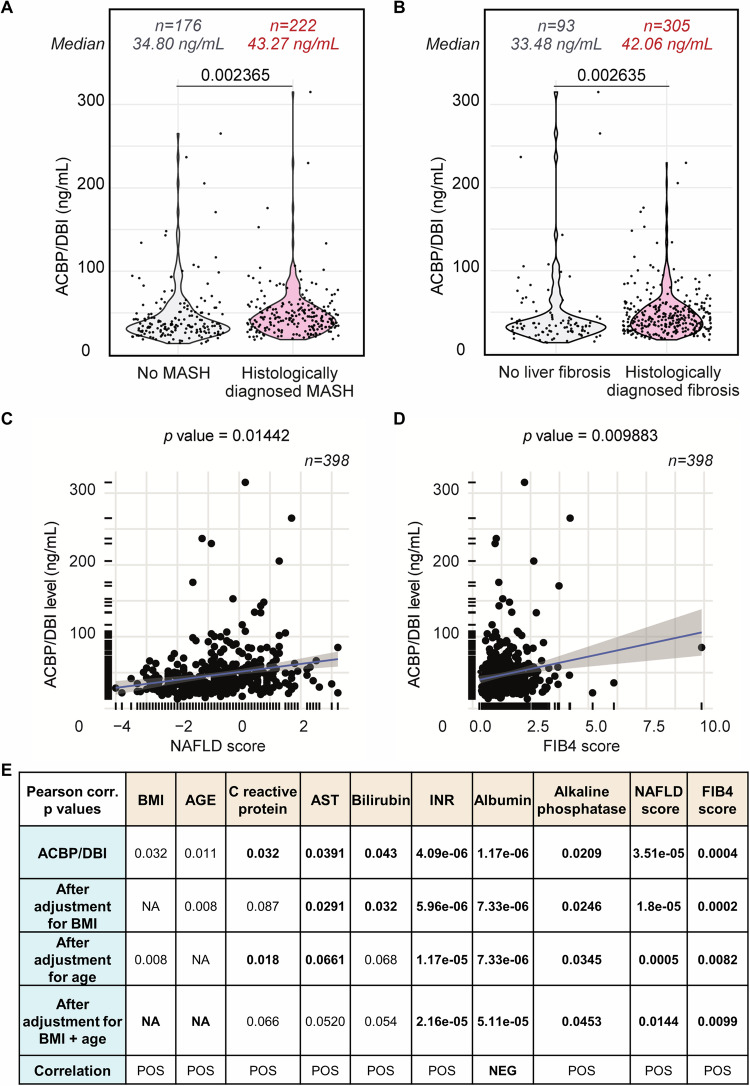
High ACBP/DBI levels in plasma from MASH patients. ACBP/DBI overabundance in patient diagnosed histologically with MASH (**A**) and fibrosis (**B**) shown as violin plots. Statistical comparisons were performed by means of the two-tailed Student**’**s t-test. Positive Spearman correlations of ACBP/DBI level with the backscatter scores for NAFLD (BS_SC NAFLD) (**C**) and for liver fibrosis (BS_SC FIB4) (**D**). ACBP/DBI correlated with several clinical parameters such as body mass index (BMI), age, C reactive protein (CRP), AST (aspartate transaminase), bilirubin, international normalized ratio (INR), albumin, alkaline phosphatase, NAFLD score, and FIB4 score (**E**).

We measured ACBP/DBI in a cohort of 398 patients with suspected metabolic dysfunction associated with steatotic liver disease (MASLD) (Supplementary Table 1, Fig. S1). Patients with histologically confirmed metabolic dysfunction associated steatohepatitis (MASH) in liver biopsies exhibited higher ACBP/DBI plasma concentrations than those lacking MASH (Fig. 1A). Similarly, histologically confirmed fibrosis was associated with elevated ACBP/DBI levels (Fig. 1B). ACBP/DBI plasma concentrations also correlated with two clinical scores measuring the severity of liver disease. This applies to the NAFLD score [31], a composite score of clinical parameters (age, body mass index [BMI], diabetes) and laboratory parameters (alanine transaminase [ALT], aspartate transaminase [AST], albumin concentrations, platelet count) (Fig. 1C), as well as to the FIB4 score [32], which only incorporates age, ALT, AST and platelet count (Fig. 1D).

It is important to note that, as previously described [33], ACBP/DBI levels correlate with age and BMI in this cohort (Fig. 1E). However, the correlations with histological signs of liver disease (Fig. 1A, B) or clinical scores (Fig. 1C, D) remained significant after adjustment for age and BMI (Fig. 1A–E). ACBP/DBI plasma levels also correlated with individual laboratory parameters indicating inflammation (C-reactive protein), hepatocyte damage (AST), three signs of liver dysfunction (high bilirubin; high international normalized ratio [INR], which is elevated when blood clotting is slowed; low albumin), as well as with serum alkaline phosphatase (which increases in hepatitis and cirrhosis) [34]. Of note, the positive correlations with INR and serum alkaline phosphatase, as well as the negative correlation with albumin, remained significant after adjustment for age and BMI (Fig. 1E).

Altogether these clinical correlations suggest that elevated plasma ACBP/DBI is a biomarker of MASH. We therefore decided to investigate the role of ACBP/DBI in preclinical models of active fatty liver disease, hence administering a neutralizing anti-ACBP/DBI mAb to mice with manifest MASH and/or fibrosis.

### Anti-ACBP/DBI mAb attenuates MASH induced by a methionine/choline-deficient diet

**Fig. 2:**
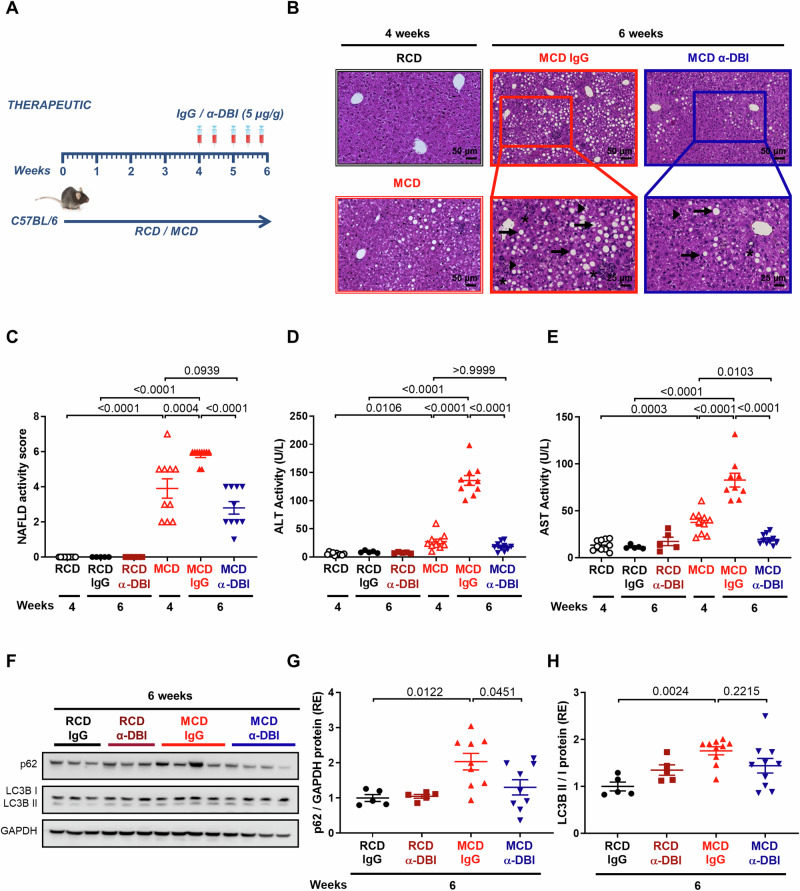
ACBP/DBI neutralization in vivo protects against MCD-induced hepatic steatosis, inflammation, and ballooning in curative setting. **A** Experimental strategy of the MASH injury induced by MCD for 4 weeks (control with regular chow diet [RCD]) in C57BL/6 mice. The diet was maintained for 2 weeks and α-DBI or IgG (5 μg/g B.W.) was administrated 1 d before week 4 and biweekly during the diet. **B**–**H** α-DBI attenuates MASH produced by MCD diet through autophagy activation. Hepatic HES images (**B**), NAFLD activity score (**C**), ALT (**D**) and AST (**E**) activity in plasma from mice treated with α-DBI or IgG. Representative Western blots (**F**) and densitometric analysis of p62 (G) normalized against GAPDH and the ratio LC3B II/I (**H**) (both expressed as relative expression, RE) from liver extracts (*n* = 5–10 mice per group). Inflammation foci, macrosteatosis, and microsteatosis vesicular were indicated as asterisk, arrows and arrowheads, respectively. Data are represented as means ± SEM. Statistical analyses (*p* values) were calculated by ANOVA test.

Mice fed a methionine/choline-deficient diet (MCD) for 4 weeks exhibit histological signs of NAFLD, mostly MASH without fibrosis (Fig. 2A–C), coupled to a modest increase in ALT and AST levels (Fig. 2D, E), meaning that the disease process is active. At this point, we started injections of anti-ACBP/DBI mAb or as a control an isotype (IgG2a)-matched antibody (IgG) while continuing the MCD until week 6. Comparison of these two groups demonstrated a strong NAFLD-inhibitor effect of ACBP/DBI neutralization as well as a reduction of ALT and AST levels (Fig. 2B–E). NAFLD activity as well as ALT + AST concentrations increased from weeks 4–6 in the control groups but were stopped in their progression if ACBP/DBI was inhibited during the last 2 weeks of the experiment (Fig. 2C–E). Of note, AST levels measured at 6 weeks of MCD diet combined with anti-ACBP/DBI were significantly lower than at 4 weeks, indicating that ACBP/DBI neutralization could normalize this parameter to control levels (Fig. 2E).

**Fig. 3:**
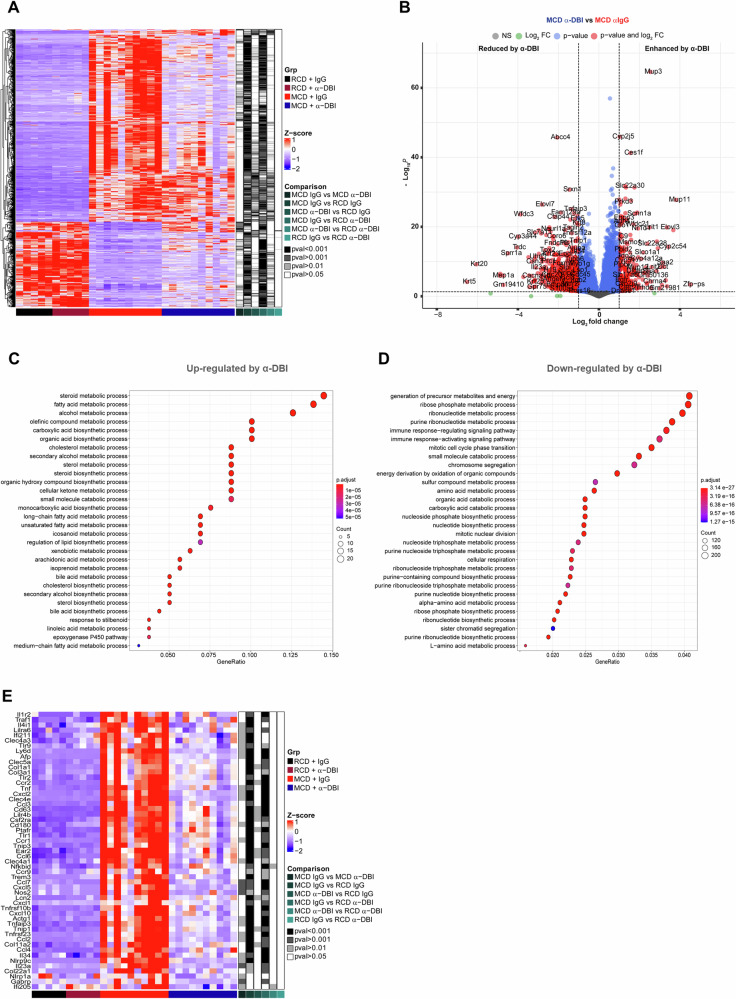
α-DBI therapeutic administration promotes a differential hepatic gene expression profile from whole transcriptome sequencing. Heat map of the hierarchical clustering of genes with fold change ≥1.0 or ≤-1.0 and *p* < 0.05) using Z score for normalized value from mice fed with RCD or MCD and treated with IgG or α-DBI (**A**). Expression level represented as Volcano plot between mice treated with α-DBI versus IgG and fed with MCD (**B**) (n = 10 mice in each group). GSEA-based KEGG pathway enrichment analysis from up-regulated genes (**C**) and downregulated genes (**D**) after α-DBI administration therapeutically. The top most significant pathways are shown in the figures. **E** Heat map of the hierarchical clustering from liver (genes manually curated involved in fibrosis and immune pathways with fold change ≥ ±1.0 and with *p* < 0.05) using Z scores for normalized value from mice fed with RCD or MCD and treated with IgG or α-DBI. Statistical analyses (*p* value) were calculated using Dunn’s test with Bonferroni correction.

Anti-ACBP/DBI reduced the liver concentrations of the autophagic substrate sequestosome-1 (STQM1, best known as p62) (Fig. 2F–H), in line with the well-known autophagy-stimulatory action of this antibody [8, 10, 14]. In addition, ACBP/DBI inhibition increases the abundance of multiple MASH-associated bile acid metabolites (Fig. S2). RNA sequencing (RNAseq) revealed that ACBP/DBI inhibition for two weeks did not induce major changes in the hepatic transcriptome in mice receiving a regular chow diet (RCD). MCD administered for 6 weeks led to the up- or down-regulation of thousands of genes. These MASH-associated shifts in the transcriptome were largely attenuated in mice receiving anti-ACBP/DBI mAb during the last 2 weeks of the MCD diet (Fig. 3A, B). Gene ontology analyses indicated a major effect of ACBP/DBI neutralization on mRNAs involved in metabolic processes, with a reduction of fibrosis and immune response-relevant mRNAs (Fig. 3C–E).

In conclusion, it appears that ACBP/DBI neutralization can halt (and partially reverse) active MASH in the MCD model even if is administered during an active phase of the disease.

### Anti-ACBP/DBI mAb reduces MASH and fibrosis induced by Western style diet alone or combined with CCl_4_

**Fig. 4:**
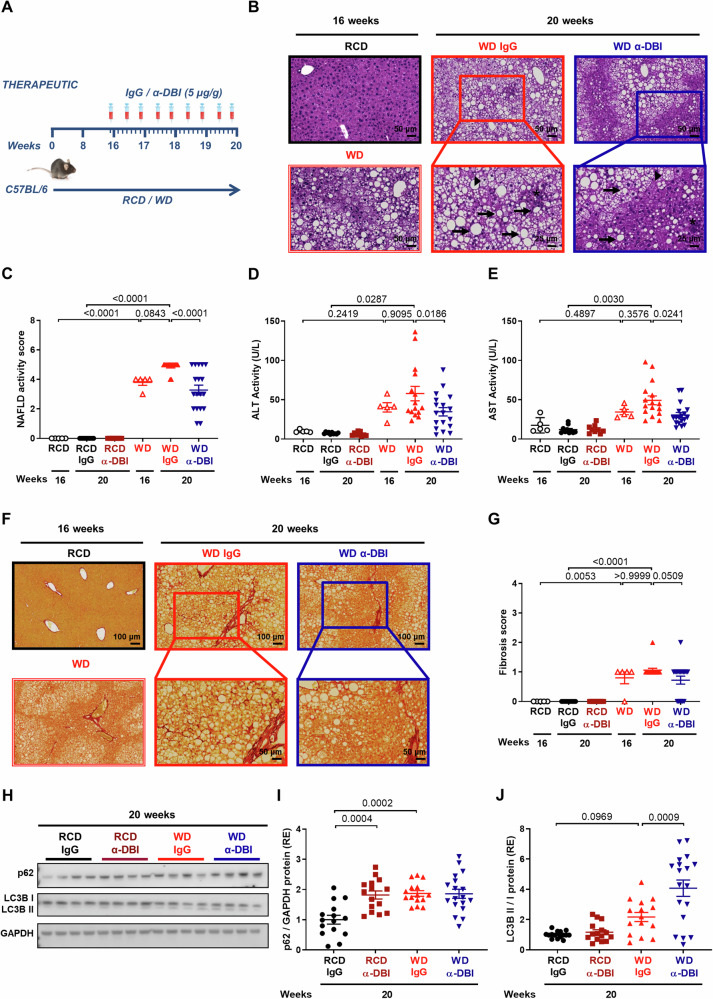
Therapeutic neutralization of ACBP/DBI attenuates MASH induced by Western diet. Schematic diagram of hepatic MASH induced by Western-style diet (WD) in C57BL/6 mice for 16 weeks. The diet was kept for 4 weeks and several doses of IgG or α-DBI (5 µg/g B.W.) was administrated from week 16 to 20 (**A**). **B**–**J** α-DBI reduces WD derived-MASH and fibrosis phenotype. Representatives HES images from liver (**B**), NAFLD activity score (**C**), ALT (**D**) and AST activity (**E**) in plasma, representative pictures of liver fibrosis (**F**), fibrosis score (**G**), and Immunoblots (**H**) and densitometric analysis of p62 (**I**) normalized against GAPDH and the ratio LC3B II/I (**J**) (both expressed as RE) from liver extracts. (n = 5–18 mice/group). Inflammation foci, macrosteatosis, and microsteatosis vesicular were indicated as asterisk, arrows, and arrowheads, respectively. Data are displayed as means ± SEM. Statistical analyses (*p* values) were calculated by ANOVA test.

In the next series of experiments, we fed mice with a high-fat, high-fructose, high-glucose, high-sucrose “Western style” diet (WD) for 16 weeks, hence inducing manifest MASH (Fig. 4A, B) with an elevation of transaminases (Fig. 4D, E) and signs of fibrosis (Fig. 4F, G). When WD was continued for additional 4 weeks in combination with IgG control injections, the disease progressed, leading to manifest fibrosis (Fig. 4F, G). Treatment during this final period with anti-ACBP/DBI mAb significantly (p < 0.05, Anova test with post-hoc Bonferroni correction) attenuated histological MASLD activity (Fig. 4B, C), ALT and AST (Fig. 4D, E) and tended (p = 0.0509) to reduce fibrosis as well (Fig. 4F, G). In the context of WD, anti-ACBP/DBI increased the lipidation of microtubule-associated proteins 1 A/1B light chain 3B (hereafter referred to as LC3) as a sign of autophagy, but failed to reduce the abundance of the autophagic substrate sequestosome-1 (SQSTM1, best known as p62) in the liver (Fig. 4H–J).

**Fig. 5:**
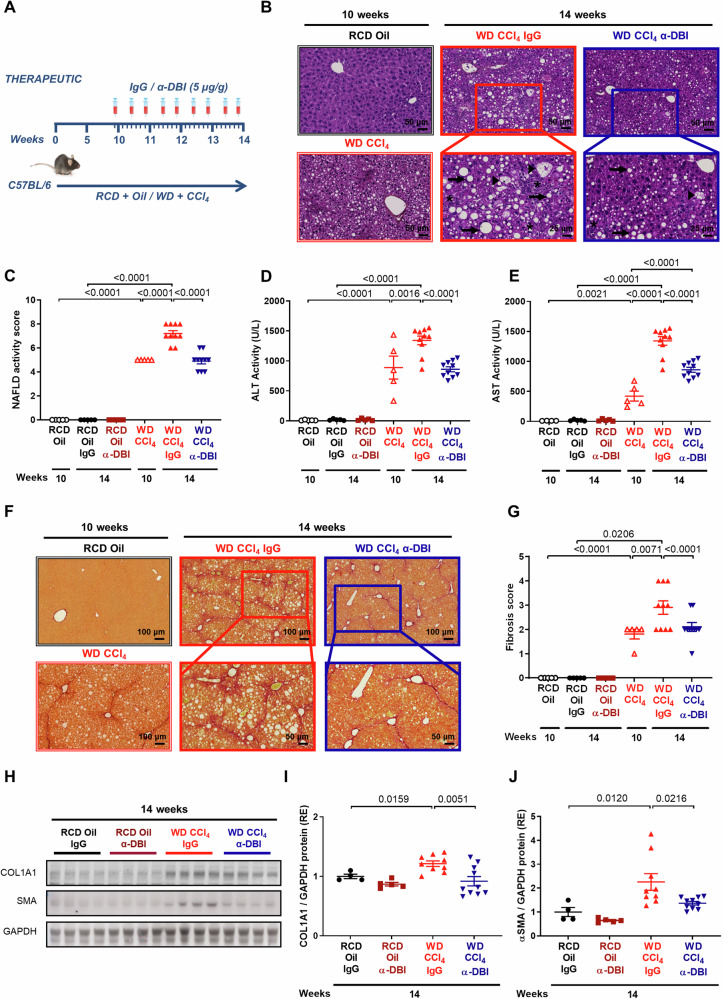
The phenotypic derived from Western diets with CCl4 is ameliorated by ACBP/DBI neutralization in vivo treatment. Schematic diagram of hepatic MASH induced by WD plus CCl_4_ in C57BL/6 mice for 10 weeks. The diet plus CCl_4_ injection was maintained for 4 weeks and several doses of IgG or α-DBI (5 µg/g) was administrated from week 10 to 14 (**A**). **B**–**J** α-DBI diminished the steatosis, inflammation, ballooning and fibrosis in vivo. Representative pictures of hepatic HES (**B**), NAFLD activity score (**C**), ALT (**D**) and AST (**E**) activity in plasma, representative images of liver fibrosis by Sirius Red (**F**), fibrosis score (**G**), and of Western Blotting (**H**) and densitometry analysis of Collagen 1A1 (**I**) and α-SMA (**J**) normalized against GAPDH (both expressed as relative expression, RE) from liver extracts. (n = 5–10 mice/group). Inflammation foci, ballooning and steatosis vesicular are indicated as asterisk, arrows, and arrowheads, respectively. The results are shown as means ± SEM. Statistical analyses (*p* values) were calculated by ANOVA test.

Next, we combined WD with weekly intraperitoneal (i.p.) injection of the hepatoxic agent CCl_4_, a protocol that causes accelerated MASLD [24] (Fig. 5A). At 10 weeks of WD+CCl_4_, mice exhibited advanced MASLD (Fig. 5B, C), major increases in ALT and AST (Fig. 5D, E), as well as liver fibrosis (Fig. 5F, G). The hepatotoxic treatment (WD+CCl_4_) was continued for another 4 weeks during which either the control IgG or anti-ACBP/DBI mAb was injected. In the absence of ACBP/DBI neutralization, MASLD activity (Fig. 5B, C), ALT and AST (Fig. 5D, E) and fibrosis (Fig. 5F, G) progressed significantly (p < 0.05, Anova test with post-hoc Bonferroni correction), and this progression was averted by anti-ACBP/DBI mAb (Fig. 5B–G). ACBP/DBI neutralization reduced the hepatic abundance of the two fibrosis markers collagen 1A1 (COL1A1) and α-smooth muscle actin (αSMA) (Fig. 5H–J), as well that of p62, likely reflecting autophagy induction (Fig. S4C, D). Moreover, ACBP/DBI inhibition caused favorable shifts in the hepatic metabolome with an increase in bile acids in liver and plasma (Fig. S3).

In sum, ACBP/DBI neutralization can avert the progression of active liver disease induced by WD alone or by the combination of WD with CCl_4_.

### Anti-ACBP/DBI mAb reduces liver damage induced by ethanol combined with CCl4

**Fig. 6:**
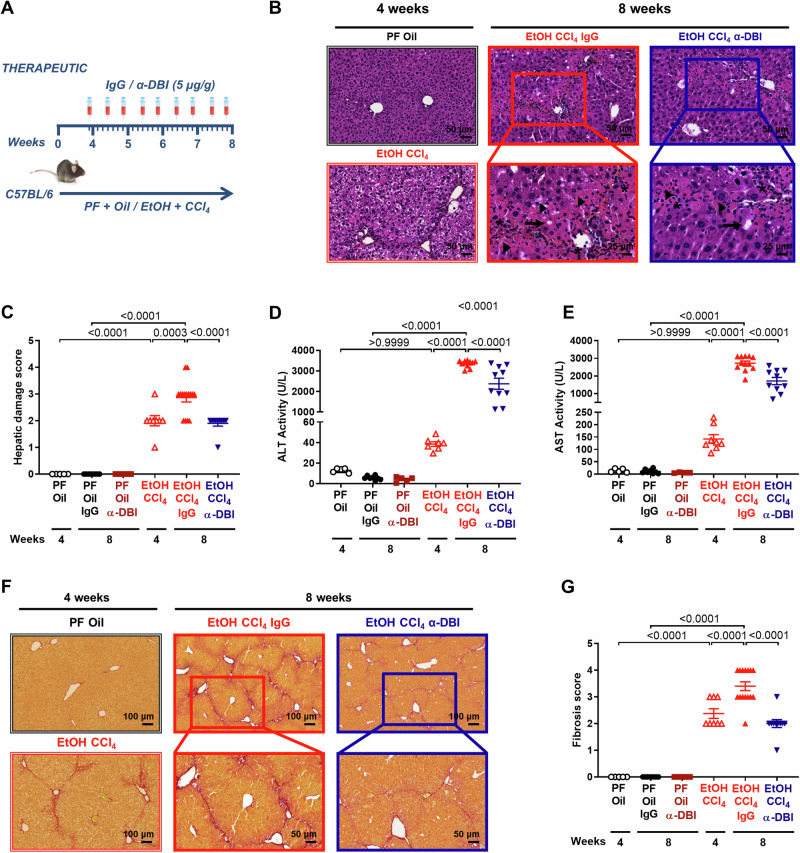
α-ACBP/DBI treatment diminishes the liver damage and fibrosis produced by Ethanol combined with CCl_4_. Schematic diagram of hepatic injury induced by Ethanol diet plus CCl_4_ in C57BL/6 mice for 4 weeks. The ethanol diet plus CCl_4_ administration was maintained for 4 more weeks and repeated doses of IgG or α-DBI (5 µg/g B.W.) were administrated from weeks 4 to 8 (**A**). **B**–**J** ACBP/DBI neutralization reduces liver damage and fibrosis in vivo. Representatives HES images from liver (**B**), hepatic damage score (**C**), plasmatic ALT (**D**) and AST activity (**E**), representative hepatic fibrosis images by Sirius Red (**F**), and fibrosis score (**G**). Inflammation foci, necrotic cell and steatosis are indicated as asterisk, arrows, and arrowheads, respectively. The results are represented as means ± SEM. Statistical analyses (*p* values) were calculated by ANOVA test.

Although MASLD had been thought to be fundamentally different in its pathogenesis from alcohol-induced liver disease, recent findings suggest that the microbiota-mediated conversion of dietary glucose into ethanol contributes to MASLD [17]. As a result, we evaluated the capacity of anti-ACBP/DBI to block active liver disease induced by continuous oral ethanol administration with a liquid diet combined by i.p. injections of CCl_4_ (Fig. 6A). After 4 weeks of ethanol+CCl_4_ treatment, the livers exhibited histological signs of damage including necrosis and cellular vacuolization (Fig. 6B, C) coupled to an increase in plasma ALT and AST (Fig. 6D, E). In addition, the liver was affected by fibrosis (Fig. 6F, G). These alterations were magnified by continuing ethanol+CCl_4_ treatment for another 4 weeks in the absence of ACBP/DBI inhibition (Fig. 6A–G). The aggravation of liver disease was attenuated by ACBP/DBI neutralization at the level of circulating transaminases, which were partially but significantly reduced (Fig. 6D, E), and fully blocked at the levels of histologically detectable damage (Fig. 6B, C) and fibrosis (Fig. 6F, G).

In this ASH model of accelerated liver damage, ACBP/DBI neutralization caused an increase in bile acids in liver and plasma (Fig. S4).

We conclude that active liver disease of rather different etiologies can be improved by ACBP/DBI neutralization even when the damaging agents (MCD, WD, WD + CCl_4_ or ethanol + CCl_4_) continue to be administered.

## Discussion

In the present paper, we provide data suggesting a role for ACBP/DBI in the pathogenesis of human MASLD. Although ACBP/DBI plasma concentrations increase with age and BMI, which both are risk factors for MASLD [21, 35], it appears that ACBP/DBI levels are particular high in patients with histologically diagnosed MASLD or liver fibrosis, correlating with various laboratory parameters of liver inflammation (CRP), insufficiency (high bilirubin and INR, low albumin) and damage (AST, alkaline phosphatase). Importantly, these associations appear independent from age and BMI, arguing in favor of the possible use of ACBP/DBI as a biomarker of liver disease. In mice, some conditions that induce MASLD (such as high-fat diet and treatment with the thiazolidinedione antidiabetic rosiglitazone) upregulate *Dbi* mRNA in the liver, but MCD fails to do so [10]. Hence, it would be interesting to correlate *Dbi* mRNA or protein levels in liver biopsies with ELISA-detectable circulating ACBP/DBI protein on a patient-by-patient basis. Unfortunately, the diagnostic liver biopsies performed in this study were not suitable for this type of analysis.

Although correlations between plasma ACBP/DBI levels and liver disease in patients cannot inform about cause-effect relationship, our preclinical data strongly support the idea that ACBP/DBI participates to the pathogenesis of NAFLD. Indeed, we have shown in the past that ACBP/DBI inhibition by various methods including mAb-mediated neutralization has hepatoprotective effects in mice against a variety of acute insults such as intoxication with paracetamol, ischemia-reperfusion damage and bile duct ligation [10]. We also showed that anti-ACBP/DBI mAb can prevent chronic liver damage by MCD, HFD or repeated CCl_4_ injections [8, 10] Here, we demonstrate that, beyond these preventive effects, ACBP/DBI neutralization can halt ongoing deterioration of the liver even when the damaging agents continue to be administered, as shown in 4 distinct models (MCD, WD, WD+CCl_4_ or ethanol +CCl_4_).

The broad hepatoprotective effects of anti-ACBP/DBI mAb appear remarkable because the mechanisms accounting for liver damage in these 4 models are rather disparate. MCD causes MASH because of an inhibition of β-oxidation, as well as because of reduced production of very low-density lipoprotein (VLDL), resulting in hepatocyte accumulation of free fatty acids and triglycerides, respectively [36]. WD causes MASLD because of the saturation of hepatocytes by lipids and sugars transported by the portal circulation [37]. Ethanol is metabolized to acetaldehyde, a potential carcinogen that causes oxidative stress and mitochondrial dysfunction but also serves as a precursor for lipogenesis [38]. WD with its excess of carbohydrates can cause the production of ethanol by fermentation by fungi and bacteria in the gut, and ethanol then reaches the liver via the portal vein [17, 39]. The hepatotoxicity of both WD and ethanol is exacerbated by co-treatment with CCl_4_, an apolar solvent that is metabolized by cytochrome P450 2E1 to generate free radicals, ultimately causing local oxidative damage [24]. Although the pathomechanisms of liver damage studied here are rather heterogeneous, ACBP/DBI neutralization provides hepatoprotection in all these models. Broad evidence in favor of hepatoprotection by ACBP/DBI neutralization is also observed at the methodological levels. Thus, different approaches, including HE and Sirius red staining of liver sections, quantitation of circulating liver enzymes, metabolomics and transcriptomics, convergently demonstrate that anti-ACBP/DBI mAb reduces all signs of liver damage, even if the disease process has already started.

Mechanistically, we have shown in the past that ACBP/DBI inhibition results in an increase in autophagic flux, which is well-known to provide protection to hepatocytes against unwarranted cell death [40], to favor the catabolism of lipids (lipophagy) and the elimination of damaged mitochondria (mitophagy) and to sequester pro-inflammatory protein complexes, thus intercepting pro-inflammatory and pro-fibrotic circuitries [8, 41]. Indeed, we previously described that ACBP/DBI neutralization induces autophagy in hepatocytes, both in vivo (in mice) [8, 10, 15] and ex vivo/in vitro (in isolated primary liver cells) [9]. We have also shown that the induction of autophagy was necessary to confer anti-ACBP/DBI-induced hepatoprotection against a variety of dietary insults [10, 15]. Indeed, autophagy inhibition by knockout of Atg4b or injection of high-dose 3-hydroxychloroquine abolished the capacity of anti-ACBP/DBI mAb to protect the liver against HFD, MCD or insult by acetaminophen, concanavalin A, CCl_4_, ischemia-reperfusion and bile duct ligation [10, 15]. It appears plausible that the obligatory implication of autophagy enhancement by ACBP/DBI neutralization in the prevention of hepatic damage can be extrapolated to the therapeutic setting as well. That said, the precise mechanisms that link induction of autophagy to hepatoprotection require further investigation.

As shown here, ACBP/DBI neutralization did induce signs of autophagy such as a reduction of p62 and/or an increase in the lipidation of LC3. Hence, it appears plausible that the observed hepatoprotective effects of anti-ACBP/DBI mAb are secondary to an increase in autophagic flux, not only in the prophylactic but also in the therapeutic setting. Lipophagy has been involved in the suppression of MALFD [42]. However, in this study we have not directly explored the role of lipophagy in the MALFD-therapeutic action of ACBP/DBI inhibition. Future studies must address this question. In the past we described that ACBP/DBI has orexigenic effects [12], meaning that its inhibition can reduce food intake and control weight gain [11, 43]. However, this mechanism likely cannot be (solely) responsible for the favorable impact of ACBP/DBI neutralization on fatty liver disease because ACBP/DBI neutralization inhibits MASH in the context of MCD, which is tied to weight loss [44] rather than weight gain, as this is the case in the context of WD [45]. Unfortunately, we did not monitor food intake in the experiments described in this paper, which constitutes one of the limitations of our study. Future investigation should elucidate to which extent the hepatoprotective effects of ACBP/DBI neutralization depend on reduced caloric intake.

In sum, we show that ACBP/DBI plasma levels are particularly high in patients with MASH and hepatic fibrosis, pointing to a potential pathogenic role of this protein in liver disease. Accordingly, we show in various preclinical models that the progression of active MASH and fibrosis can be attenuated by injection of a neutralizing mAb specific for ACBP/DBI. Future studies must address the possibility to translate these findings to the clinics.

## Supplementary information

Supplementary Materials

Supplemental Table 1

Raw_WB

## Data Availability

The data that support the findings of this study are available from the corresponding authors upon reasonable request. Metabolomics data are available at Mendeley data 10.17632/vv564y44mm.1. Transcriptomics data are available at https://www.ncbi.nlm.nih.gov/geo/as GSE278209.

## References

[1] Tonon MC, Vaudry H, Chuquet J, Guillebaud F, Fan J, Masmoudi-Kouki O, et al. Endozepines and their receptors: Structure, functions and pathophysiological significance. Pharmacol Ther. 2020;208:107386. 31283949 10.1016/j.pharmthera.2019.06.008

[2] Alquier T, Christian-Hinman CA, Alfonso J, Faergeman NJ. From benzodiazepines to fatty acids and beyond: revisiting the role of ACBP/DBI. Trends Endocrinol Metab. 2021;32:890–903. 34565656 10.1016/j.tem.2021.08.009 PMC8785413

[3] Montegut L, Abdellatif M, Motino O, Madeo F, Martins I, Quesada V, et al. Acyl coenzyme A binding protein (ACBP): An aging- and disease-relevant “autophagy checkpoint”. Aging Cell. 2023;22:e13910. 37357988 10.1111/acel.13910 PMC10497816

[4] Rasmussen JT, Rosendal J, Knudsen J. Interaction of acyl-CoA binding protein (ACBP) on processes for which acyl-CoA is a substrate, product or inhibitor. Biochem J. 1993;292:907–13. 8318018 10.1042/bj2920907 PMC1134200

[5] Duman C, Yaqubi K, Hoffmann A, Acikgoz AA, Korshunov A, Bendszus M, et al. Acyl-CoA-binding protein drives glioblastoma tumorigenesis by sustaining fatty acid oxidation. Cell Metab. 2019;30:274–89.e5. 31056285 10.1016/j.cmet.2019.04.004

[6] Christian CA, Herbert AG, Holt RL, Peng K, Sherwood KD, Pangratz-Fuehrer S, et al. Endogenous positive allosteric modulation of GABA(A) receptors by diazepam binding inhibitor. Neuron. 2013;78:1063–74. 23727119 10.1016/j.neuron.2013.04.026 PMC3987987

[7] Dumitru I, Neitz A, Alfonso J, Monyer H. Diazepam binding inhibitor promotes stem cell expansion controlling environment-dependent neurogenesis. Neuron. 2017;94:125–37.e5. 28343864 10.1016/j.neuron.2017.03.003

[8] Bravo-San Pedro JM, Sica V, Martins I, Pol J, Loos F, Maiuri MC, et al. Acyl-CoA-binding protein is a lipogenic factor that triggers food intake and obesity. Cell Metab. 2019;30:754–67.e9. 31422903 10.1016/j.cmet.2019.07.010

[9] Anagnostopoulos G, Saavedra E, Lambertucci F, Motino O, Dimitrov J, Roiz-Valle D, et al. Inhibition of acyl-CoA binding protein (ACBP) by means of a GABA(A)Rgamma2-derived peptide. Cell Death Dis. 2024;15:249. 38582872 10.1038/s41419-024-06633-6 PMC10998878

[10] Motino O, Lambertucci F, Anagnostopoulos G, Li S, Nah J, Castoldi F, et al. ACBP/DBI protein neutralization confers autophagy-dependent organ protection through inhibition of cell loss, inflammation, and fibrosis. Proc Natl Acad Sci USA. 2022;119:e2207344119. 36191214 10.1073/pnas.2207344119 PMC9565466

[11] Joseph A, Moriceau S, Sica V, Anagnostopoulos G, Pol J, Martins I, et al. Metabolic and psychiatric effects of acyl coenzyme A binding protein (ACBP)/diazepam binding inhibitor (DBI). Cell Death Dis. 2020;11:502. 32632162 10.1038/s41419-020-2716-5 PMC7338362

[12] Chen H, Moriceau S, Joseph A, Mailliet F, Li S, Tolle V et al. Acyl-CoA binding protein for the experimental treatment of anorexia. Sci Transl Med. 2024;16:eadl0715. 10.1126/scitranslmed.adl0715 39141698

[13] Montegut L, Chen H, Bravo-San Pedro JM, Motino O, Martins I, Kroemer G. Immunization of mice with the self-peptide ACBP coupled to keyhole limpet hemocyanin. STAR Protoc. 2022;3:101095. 35059656 10.1016/j.xpro.2021.101095 PMC8760546

[14] Montegut L, Joseph A, Chen H, Abdellatif M, Ruckenstuhl C, Motino O, et al. High plasma concentrations of acyl-coenzyme A binding protein (ACBP) predispose to cardiovascular disease: evidence for a phylogenetically conserved proaging function of ACBP. Aging Cell. 2023;22:e13751. 36510662 10.1111/acel.13751 PMC9835587

[15] Motino O, Lambertucci F, Anagnostopoulos G, Li S, Martins I, Kroemer G. Cardio-, hepato- and pneumoprotective effects of autophagy checkpoint inhibition by targeting DBI/ACBP. Autophagy. 2023;19:1604–6. 36198092 10.1080/15548627.2022.2131241 PMC10240994

[16] Eslam M, Newsome PN, Sarin SK, Anstee QM, Targher G, Romero-Gomez M, et al. A new definition for metabolic dysfunction-associated fatty liver disease: an international expert consensus statement. J Hepatol. 2020;73:202–9. 32278004 10.1016/j.jhep.2020.03.039

[17] Meijnikman AS, Davids M, Herrema H, Aydin O, Tremaroli V, Rios-Morales M, et al. Microbiome-derived ethanol in nonalcoholic fatty liver disease. Nat Med. 2022;28:2100–6. 36216942 10.1038/s41591-022-02016-6

[18] Poynard T, Paradis V, Mullaert J, Deckmyn O, Gault N, Marcault E, et al. Prospective external validation of a new non-invasive test for the diagnosis of non-alcoholic steatohepatitis in patients with type 2 diabetes. Aliment Pharmacol Ther. 2021;54:952–66. 34398492 10.1111/apt.16543 PMC8518776

[19] Poynard T, Deckmyn O, Peta V, Paradis V, Gautier JF, Brzustowski A, et al. Prospective direct comparison of non-invasive liver tests in outpatients with type 2 diabetes using intention-to-diagnose analysis. Aliment Pharmacol Ther. 2023;58:888–902. 37642160 10.1111/apt.17688

[20] Dioguardi Burgio M, Castera L, Oufighou M, Rautou PE, Paradis V, Bedossa P, et al. Prospective comparison of attenuation imaging and controlled attenuation parameter for liver steatosis diagnosis in patients with nonalcoholic fatty liver disease and type 2 diabetes. Clin Gastroenterol Hepatol. 2024;22:1005–13.e27. 38072287 10.1016/j.cgh.2023.11.034

[21] Castera L, Garteiser P, Laouenan C, Vidal-Trécan T, Vallet-Pichard A, Manchon P, et al. Prospective head-to-head comparison of non-invasive scores for diagnosis of fibrotic MASH in patients with type 2 diabetes. J Hepatol. 2024;81:195–206. 10.1016/j.jhep.2024.03.023 38548067

[22] Castera L, Laouenan C, Vallet-Pichard A, Vidal-Trecan T, Manchon P, Paradis V, et al. High prevalence of NASH and advanced fibrosis in type 2 diabetes: a prospective study of 330 outpatients undergoing liver biopsies for elevated ALT, using a low threshold. Diabetes Care. 2023;46:1354–62. 37043830 10.2337/dc22-2048

[23] Li S, Motino O, Lambertucci F, Chen H, Anagnostopoulos G, Montegut L, et al. A mouse model of non-alcoholic steatohepatitis and hepatocellular carcinoma induced by western diet and carbon tetrachloride. Methods Mol Biol. 2024;2769:57–65. 38315388 10.1007/978-1-0716-3694-7_4

[24] Tsuchida T, Lee YA, Fujiwara N, Ybanez M, Allen B, Martins S, et al. A simple diet- and chemical-induced murine NASH model with rapid progression of steatohepatitis, fibrosis and liver cancer. J Hepatol. 2018;69:385–95. 29572095 10.1016/j.jhep.2018.03.011 PMC6054570

[25] Satishchandran A, Ambade A, Rao S, Hsueh YC, Iracheta-Vellve A, Tornai D, et al. MicroRNA 122, regulated by GRLH2, protects livers of mice and patients from ethanol-induced liver disease. Gastroenterology. 2018;154:238–52.e7. 28987423 10.1053/j.gastro.2017.09.022 PMC5742049

[26] Lieber CS, DeCarli LM. Quantitative relationship between amount of dietary fat and severity of alcoholic fatty liver. Am J Clin Nutr. 1970;23:474–8. 5462407 10.1093/ajcn/23.4.474

[27] Liang W, Menke AL, Driessen A, Koek GH, Lindeman JH, Stoop R, et al. Establishment of a general NAFLD scoring system for rodent models and comparison to human liver pathology. PLoS ONE. 2014;9:e115922. 25535951 10.1371/journal.pone.0115922 PMC4275274

[28] Suzuki S, Toledo-Pereyra LH, Rodriguez FJ, Cejalvo D. Neutrophil infiltration as an important factor in liver ischemia and reperfusion injury. Modulating effects of FK506 and cyclosporine. Transplantation. 1993;55:1265–72. 7685932 10.1097/00007890-199306000-00011

[29] Montegut L, Joseph A, Chen H, Abdellatif M, Ruckenstuhl C, Martins I, et al. DBI/ACBP is a targetable autophagy checkpoint involved in aging and cardiovascular disease. Autophagy. 2023;19:2166–9. 36579946 10.1080/15548627.2022.2160565 PMC10283417

[30] Alves Costa Silva C, Piccinno G, Suissa D, Bourgin M, Schreibelt G, Durand S, et al. Influence of microbiota-associated metabolic reprogramming on clinical outcome in patients with melanoma from the randomized adjuvant dendritic cell-based MIND-DC trial. Nat Commun. 2024;15:1633. 38395948 10.1038/s41467-024-45357-1 PMC10891084

[31] Mendler MH, Kanel G, Govindarajan S. Proposal for a histological scoring and grading system for non-alcoholic fatty liver disease. Liver Int. 2005;25:294–304. 15780053 10.1111/j.1478-3231.2005.01052.x

[32] Sterling RK, Lissen E, Clumeck N, Sola R, Correa MC, Montaner J, et al. Development of a simple noninvasive index to predict significant fibrosis in patients with HIV/HCV coinfection. Hepatology. 2006;43:1317–25. 16729309 10.1002/hep.21178

[33] Joseph A, Chen H, Anagnostopoulos G, Montegut L, Lafarge A, Motino O, et al. Effects of acyl-coenzyme A binding protein (ACBP)/diazepam-binding inhibitor (DBI) on body mass index. Cell Death Dis. 2021;12:599. 34108446 10.1038/s41419-021-03864-9 PMC8190068

[34] Sharma P. Value of liver function tests in cirrhosis. J Clin Exp Hepatol. 2022;12:948–64. 35677506 10.1016/j.jceh.2021.11.004 PMC9168739

[35] Hirning F. Utah impaired pharmacist program is best. Am Pharm. 1988;NS28:8. 3195468

[36] Yamaguchi K, Yang L, McCall S, Huang J, Yu XX, Pandey SK, et al. Inhibiting triglyceride synthesis improves hepatic steatosis but exacerbates liver damage and fibrosis in obese mice with nonalcoholic steatohepatitis. Hepatology. 2007;45:1366–74. 17476695 10.1002/hep.21655

[37] Parlati L, Regnier M, Guillou H, Postic C. New targets for NAFLD. JHEP Rep. 2021;3:100346. 34667947 10.1016/j.jhepr.2021.100346 PMC8507191

[38] Birková A, Hubková B, Čižmárová B, Bolerázska B. Current View on the Mechanisms of Alcohol-Mediated Toxicity. Int J Mol Sci. 2021;22:9686. 10.3390/ijms22189686 PMC8472195 34575850

[39] Bayoumy AB, Mulder CJJ, Mol JJ, Tushuizen ME. Gut fermentation syndrome: a systematic review of case reports. U Eur Gastroenterol J. 2021;9:332–42. 10.1002/ueg2.12062 PMC8259373 33887125

[40] Brenner C, Galluzzi L, Kepp O, Kroemer G. Decoding cell death signals in liver inflammation. J Hepatol. 2013;59:583–94. 23567086 10.1016/j.jhep.2013.03.033

[41] Klionsky DJ, Petroni G, Amaravadi RK, Baehrecke EH, Ballabio A, Boya P, et al. Autophagy in major human diseases. EMBO J. 2021;40:e108863. 34459017 10.15252/embj.2021108863 PMC8488577

[42] Grefhorst A, van de Peppel IP, Larsen LE, Jonker JW, Holleboom AG. The role of lipophagy in the development and treatment of non-alcoholic fatty liver disease. Front Endocrinol. 2020;11:601627. 10.3389/fendo.2020.601627 PMC7883485 33597924

[43] Charmpilas N, Ruckenstuhl C, Sica V, Buttner S, Habernig L, Dichtinger S, et al. Acyl-CoA-binding protein (ACBP): a phylogenetically conserved appetite stimulator. Cell Death Dis. 2020;11:7. 31907349 10.1038/s41419-019-2205-x PMC6944704

[44] Matthews DR, Li H, Zhou J, Li Q, Glaser S, Francis H, et al. Methionine- and choline-deficient diet-induced nonalcoholic steatohepatitis is associated with increased intestinal inflammation. Am J Pathol. 2021;191:1743–53. 34242656 10.1016/j.ajpath.2021.06.010 PMC8485057

[45] Farrell G, Schattenberg JM, Leclercq I, Yeh MM, Goldin R, Teoh N, et al. Mouse models of nonalcoholic steatohepatitis: toward optimization of their relevance to human nonalcoholic steatohepatitis. Hepatology. 2019;69:2241–57. 30372785 10.1002/hep.30333

