# Supplementary material for: ACBP/DBI neutralization for the experimental treatment of fatty liver disease
Source: Cell Death Differ. 2024 Nov 16;32(3):434–46. doi: 10.1038/s41418-024-01410-6 (PMC11894144; doi:10.1038/s41418-024-01410-6)
Supplement: Supplementary file 1 — Supplementary Materials [file 41418_2024_1410_MOESM1_ESM.docx]

**ACBP/DBI neutralization for the experimental treatment of fatty liver disease**

**Omar Motiño^1,2,3#^, Flavia Lambertucci^1,2^, Adrien Joseph^1,2^, Sylvère Durand^1,2^, Gerasimos Anagnostopoulos^1,2^, Sijing Li^1,2^, Vincent Carbonnier^1,2^, Uxía Nogueira-Recalde^1,2,4^, Léa Montégut^1,2^, Hui Chen^1,2^, Fanny Aprahamian^1,2^, Nitharsshini Nirmalathasan^1,2^, Maria Chiara Maiuri^1,2,5^, Federico Pietrocola^6^, Dominique Valla^7,8^, Cédric Laouénan^9,10^, Jean-François Gautier^11,12^, Laurent Castera^13^, QUID NASH Investigators*, Isabelle Martins^1,2#^, and Guido Kroemer^1,2, 14#^**

^1^ Centre de Recherche des Cordeliers, Inserm U1138, Université de Paris, Sorbonne Université, Equipe labellisée par la Ligue contre le cancer, Institut Universitaire de France, Paris, France.

^2^ Metabolomics and Cell Biology Platforms, Institut Gustave Roussy, Villejuif, France.

^3^ Unidad de Excelencia, Instituto de Biología y Genética Molecular (IBGM), Universidad de Valladolid - CSIC, Valladolid, Spain.

^4^ Grupo de Investigación en Reumatología (GIR), Instituto de Investigación Biomédica de A Coruña (INIBIC), Fundación Profesor Novoa Santos, A Coruña, España

^5^ Department of Molecular Medicine and Medical Biotechnologies, University of Napoli Federico II, 80131 Naples, Italy.

^6^ Department of Bioscience and Nutrition, Karolinska Institute, Huddinge, Sweden.

^7^ Université Paris Cité, Inserm U1149, Centre de recherche sur l'inflammation, F-75018, Paris, France.

^8^ Service hépatologie, AP-HP, Hôpital Beaujon, 92110, Clichy, France.

^9^ Université Paris Cité and Université Sorbonne Paris Nord, Inserm U1137, Laboratory "Infection, Antimicrobials, Modelling, Evolution" (IAME), Paris, France.

^10^ Département d'Epidémiologie Biostatistique et Recherche Clinique, AP-HP.Nord, Hôpital Bichat, Paris, France.

^11^ Institut Necker Enfants Malades, Inserm U1151, CNRS UMR 8253, IMMEDIAB Laboratory, Paris, France.

^12^ Centre Universitaire de Diabétologie et de ses Complications, AP-HP, Hôpital Lariboisière, Paris, France.

^13^ Université Paris Cité, UMR1149 (CRI), Inserm, F-75018 Paris, France ; Service d'hépatologie, AP-HP, Hôpital Beaujon, F-92110 Clichy-la-Garenne, France

^14^ Pôle de Biologie, Hôpital Européen Georges Pompidou, AP-HP, Institut Universitaire de France, Paris, France.

*****Lists of authors and their affiliations appear at the end of the paper.

^#^Correspondence: Guido Kroemer ([Kroemer@orange.fr)](mailto:Kroemer@orange.fr)), Isabelle Martins (isabelle.martins@inserm.fr) and Omar Motino ([omar.motino@uva.es](mailto:omar.motino@uva.es))

**Supplemental Figures**

**
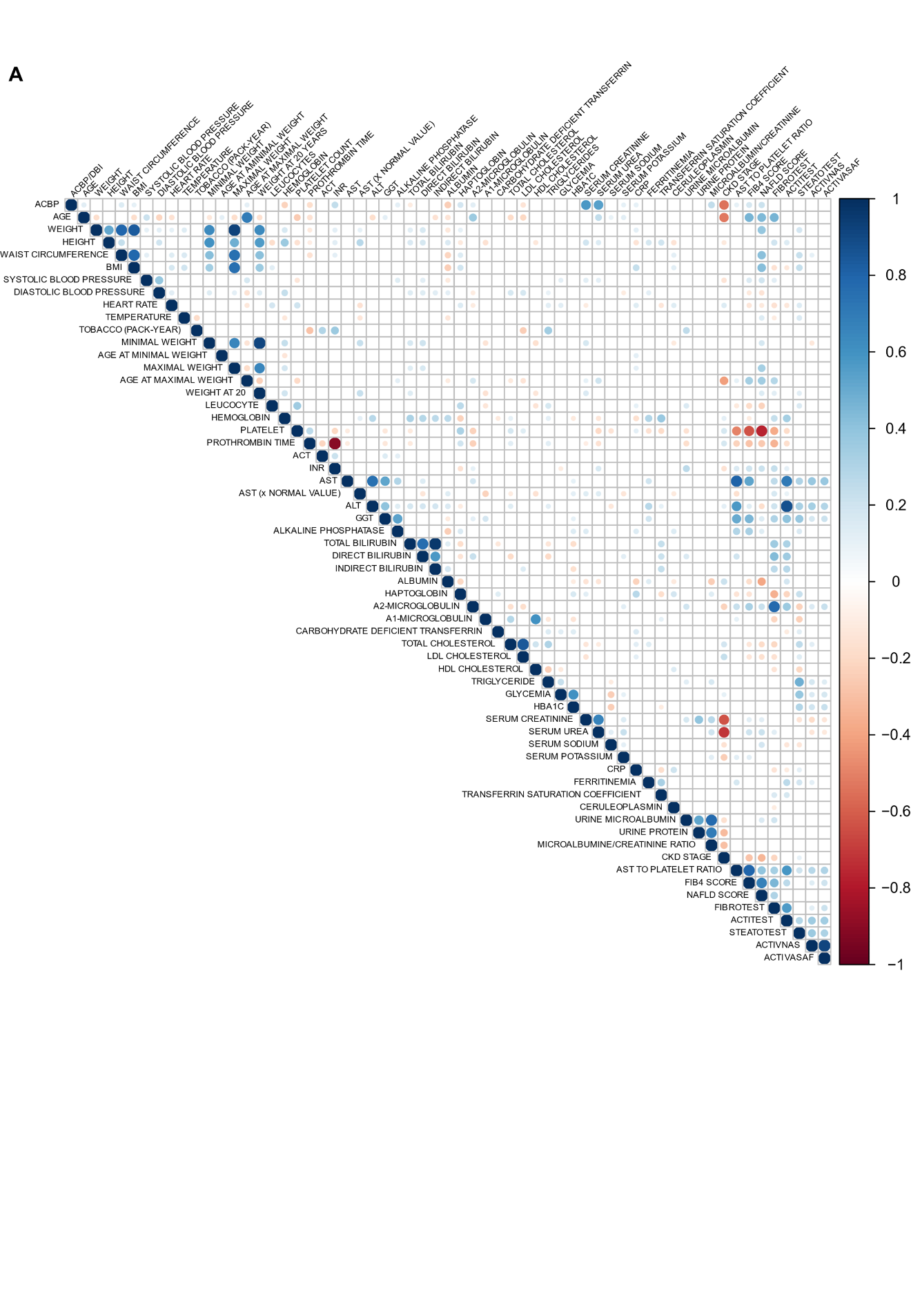
**

**Supplemental Figure 1. Correlations of ACBP/DBI levels with clinical parameters from NASH patients.** Spearman correlations are indicated.

**
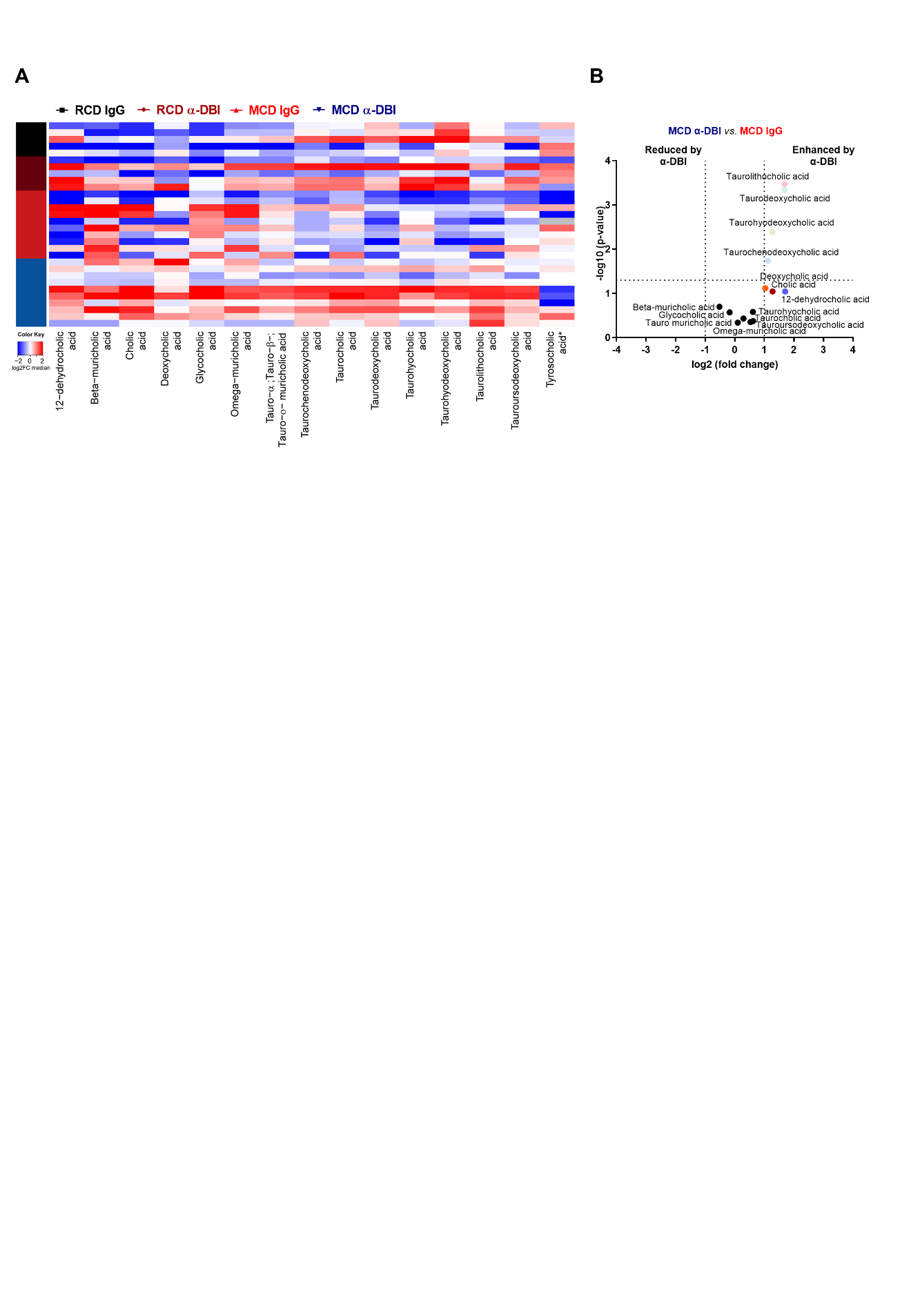
**

**Supplemental Figure 2. ACBP/DBI neutralization treatment produces changes on the profile of metabolites generated by methionine/choline deficient diet.** Heatmap clustered by Euclidean distance of changes of bile acid metabolite concentrations depicted as log2 fold change (FC) in C57BL/6 mice injected with IgG or anti-DBI after regular chow diet (RCD) or methionine/choline deficient diet **(**MCD) (n = 5–14 mice per group) (**A**). Volcano plot of bile acid metabolites from liver in MCD-treated mice treated with anti-DBI *vs.* IgG (n = 5–14 mice per group), p-values were calculated by means of a Kruskal Wallis test followed by a BH adjusted Dunn’s test. Fold changes were calculated for quality control corrected areas that were log2 transformed and mean centered (B).

The results are represented as means ± SEM. Statistical analyses (p values) were calculated by Wilcoxon (A-B), and Kruskal Wallis tests.

**
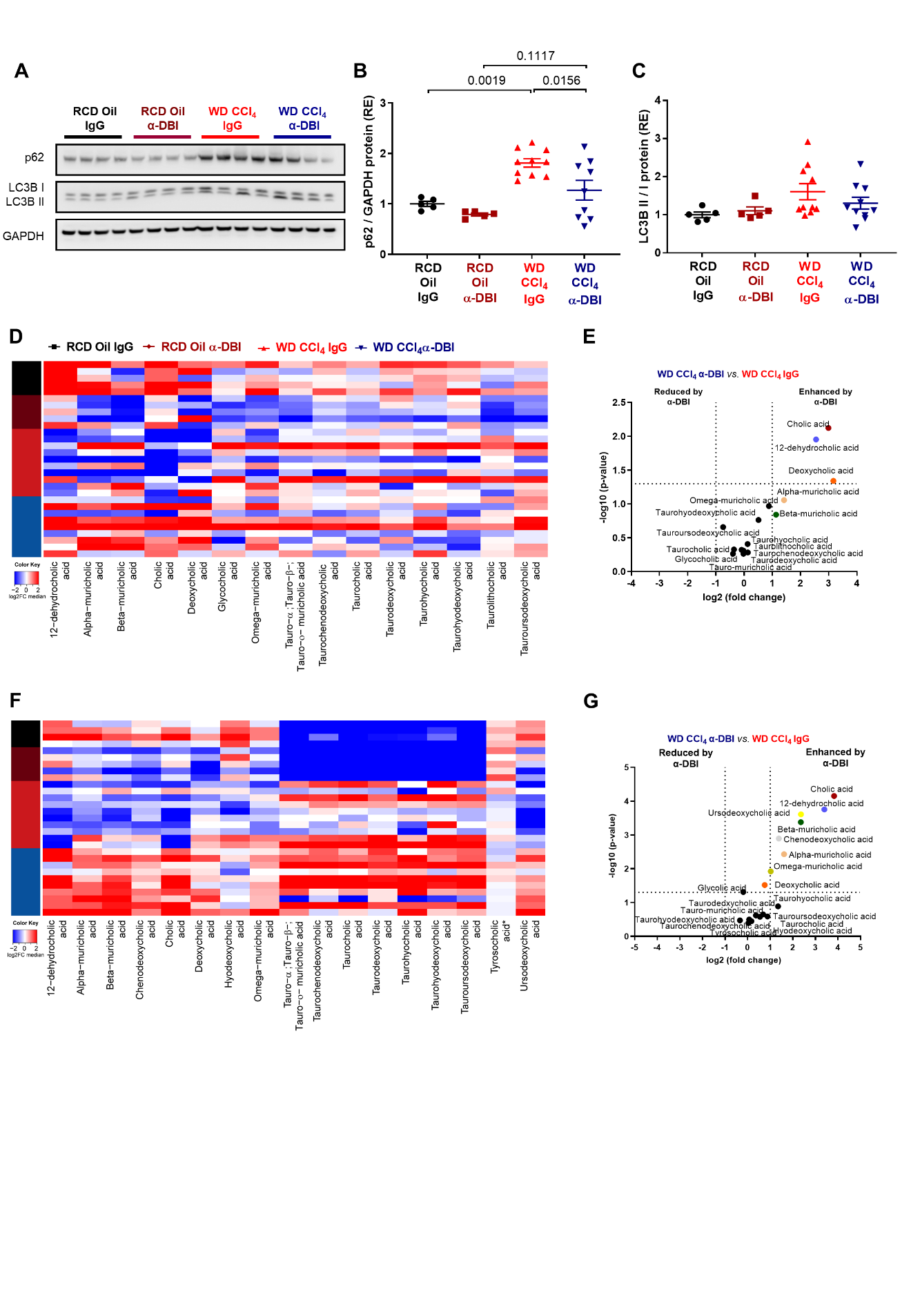
Supplemental Figure 3. The neutralization of ACBP/DBI reveals metabolic changes through autophagy activation in the context of WD diet plus CCl_4_.** Representative Western Blot (**A**) and densitometric analysis of autophagy markers as p62 (expressed as relative expression, RE) (**B**) and LC3 II/I (**C**) (represented as RE). Heatmap clustered by Euclidean distance of changes in liver (**D**) and plasma (F) of bile acid metabolite concentrations depicted as log2 fold change (FC) in C57BL/6 mice injected with IgG or anti-DBI after WD diet plus CCl_4_ (n = 5–14 mice per group). Volcano plot of bile acid metabolites from liver (**E**) and plasma (**G**) in WD diet plus CCl_4_ treated with anti-DBI *vs.* IgG (n = 5–14 mice per group).

The results are represented as means ± SEM. Statistical analyses (p value) were calculated by ANOVA tests (**B,C**) and Kruskal Wallis tests followed by Dunn’s BH adjusted test (**E,G**). Fold change values (**D,F**) are described in Fig. S2B.


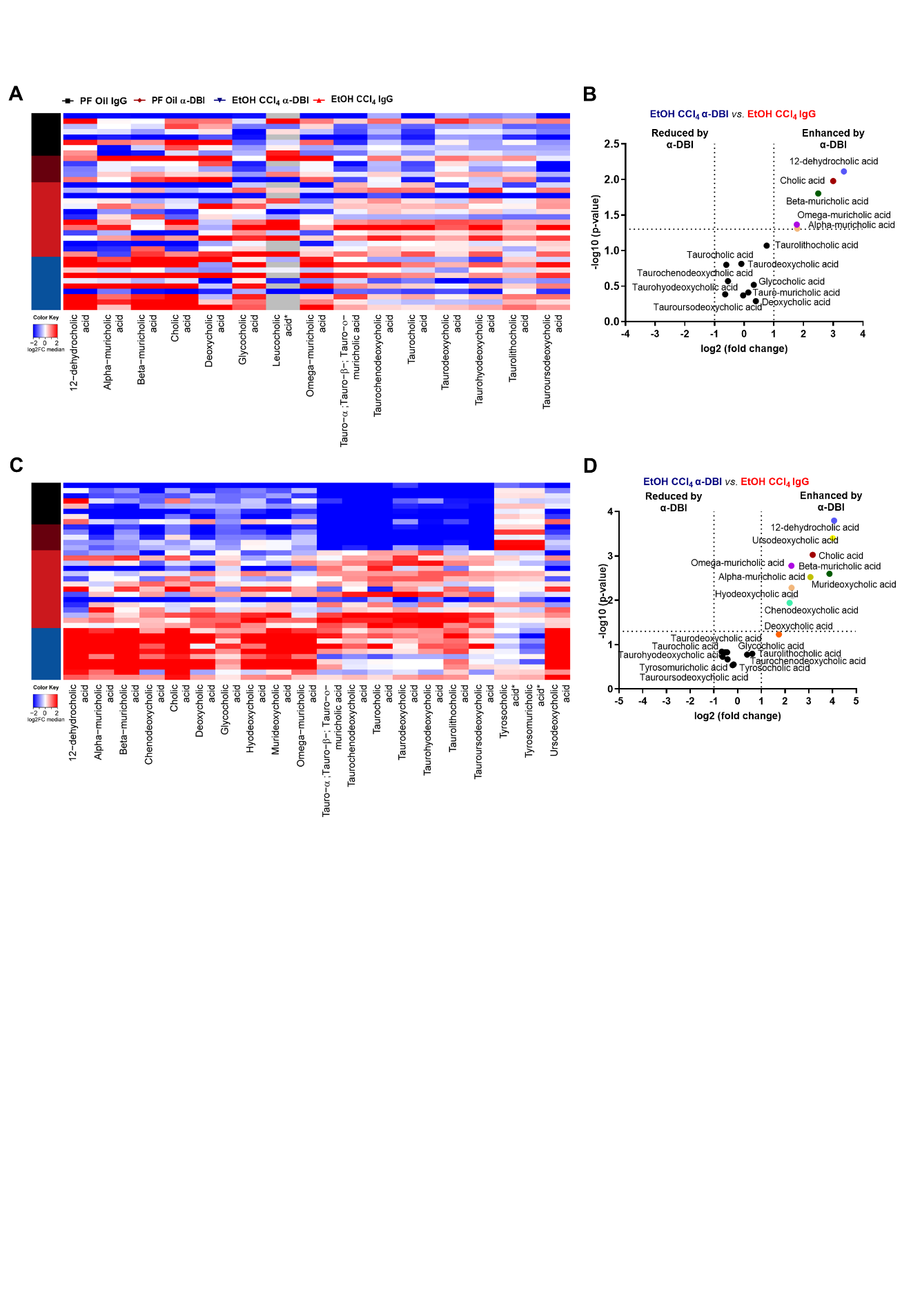


**Supplemental Figure 4. ACBP/DBI neutralization causes metabolic changes in ethanol-induced liver damage.** Heatmaps clustered by Euclidean distance of changes in liver (**A**) and plasma (**C**) bile acid metabolite concentrations depicted as log2 fold change (FC) in C57BL/6 mice injected with IgG or anti-DBI during EtOH diet plus CCl_4_ (n = 5–14 mice per group). Volcano plots of bile acid metabolites from liver (**B**) and plasma (**D**) in EtOH diet plus CCl_4_ treated with anti-DBI *vs.* IgG (n = 5–14 mice per group).

The results are represented as means ± SEM. Statistical analyses (p value) were calculated by Kruskal Wallis followed by Dunn’s BH adjusted test (**B,D**). Fold changes (**A,C**) are described in Fig. S2B.

**Full list of QUID-NASH consortium members**

**Clinical research personnel**: Djamila Bellili, Ouarda Bessadi, Charlene Da Silveira, Fatima Zohra Djelouat, Benoit Girard, Vanessa Legrand, Nathalie Neveux, Meriam Meziani, Ludovic Roy, Dahia Sekour, Manon Sens, Miassa Slimani and Ouassila Zatout.

**Département d’Epidémiologie, Biostatistiques et Recherche Clinique, HUPNVS** (Université de Paris, Assistance Publique-Hôpitaux de Paris Inserm IAME-UMR1137 and URC PNVS): Delphine Bachelet, Krishna Bhavsar, Basma Basli-Baillet Jimmy Mullaert, Estelle Marcault, and Nassima Si-Mohammed.

**Hôpital Avicenne group** (Université Paris 13, Assistance publique-Hôpitaux de Paris and Inserm U955, équipe 18, Université Paris-Est, Créteil): Emmanuel Cosson.

**Hôpital Beaujon** (Université de Paris, Assistance Publique-Hôpitaux de Paris and Inserm CRI-UMR1149): Miguel Albuquerque, Sabrina Doblas, Adel Hammoutene, Estefania Gonzalez Montpetit and Gwenaël Pagé.

**Hôpital Cochin** (Université de Paris, Assistance Publique-Hôpitaux de Paris and Institut Cochin): *centre ressources biologiques*, Béatrice Parfait; *signalisation de l’insuline et du glucose, glucotoxicité,* Catherine Postic; *immunologie du diabète,* Agnès Lehuen, Amine Toubal, Camille Rousseau, Blandine Fruchet, Pauline Soulard and Zouriatou Gouda; *génomique,* Michel Vidaud and Franck Letourneur; *imagerie du vivant*, Gilles Renault; and *régulation des cellules béta pancréatiques,* Raphaël Scharfmann.

**Hôpital Européen George Pompidou** (Université de Paris, Assistance Publique-Hôpitaux de Paris and Inserm UMR1153): *service de nutrition*, Amel Ait-Boudaoud, Charles Barsamian, Claire Carette and Claire Rives-Lange.

**Laboratoire d’Imagerie Biomédicale Sorbonne Université** (Inserm and CNRS): Rachel Baida and Olivier Couture.

**Physics for Medicine Paris** (Inserm, CNRS and Ecole supérieure de Physique et Chimie de Paris ESPCI): Sofiane Decombas, Thomas Deffieux, Thu-mai Nguyen and Mickael Tanter.

**Servier**: Tania Baltauss, Edwige-Ludiwyne Balzac, Pierre Barbier Saint Hilaire Philippe Delerive, Valérie Duvivier, Arnaud Fillon, Julia Geronimi, Jessica Laplume, Erwan Werner and Laura Xuereb.

**Swiss Institute of Bioinformatics**, **Vital-IT**: Robin Liechti, Olivier Martin, Florence Mehl and Manuela Pruess.

**BioPrédictive**: Jean-Marie Castille, Fabienne Drane, and Olivier Deckmyn.

**Commissariat à l’Energie Atomique**: Florence Castelli, Benoit Colsch Emmanuel Cousin and François Fenaille.

**Direction de la Recherche Clinique et de l’Innovation, APHP**: Laure Guilbaud and Allyre Lohier.

**Direction Régionale Inserm Paris 5 et Paris 7**: Francois Chambellin and Lyddie Laaland.

**Inserm Transfert** : Catherine Clusel, Marie Hauduroy and Pierre Pautre.
