# Supplementary material for: ACBP/DBI neutralization for the experimental treatment of fatty liver disease
Source: Cell Death Differ. 2024 Nov 16;32(3):434–46. doi: 10.1038/s41418-024-01410-6 (PMC11894144; doi:10.1038/s41418-024-01410-6)

# Figure 2

**LIVER p62**

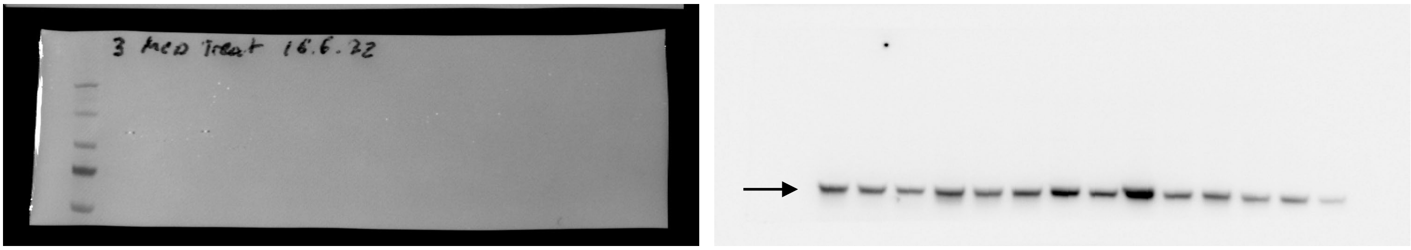

**LIVER LC3B**

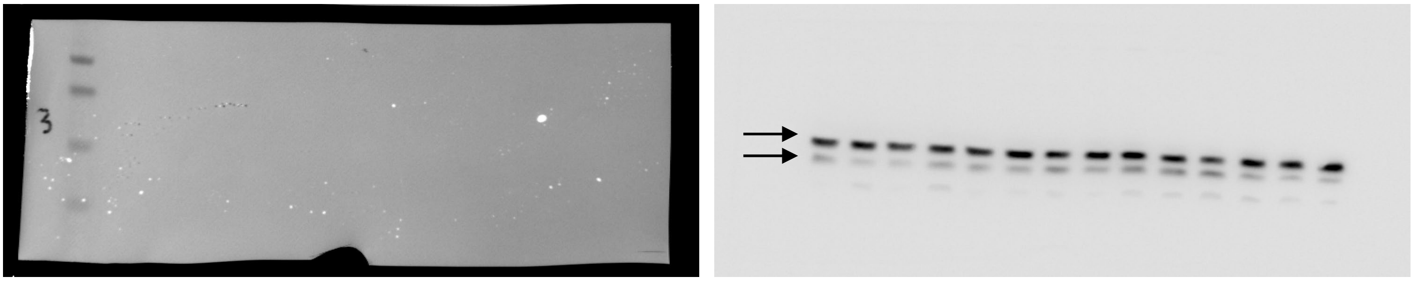

**LIVER GAPDH**

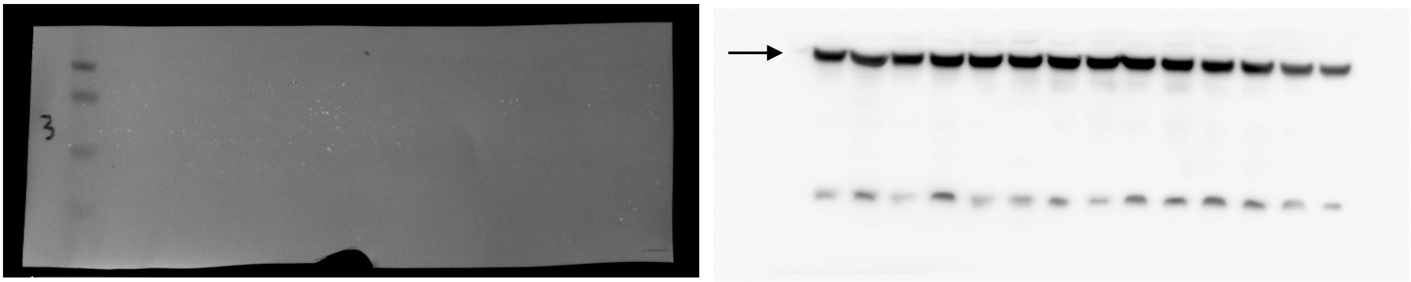

# Figure 4

**LIVER p62**

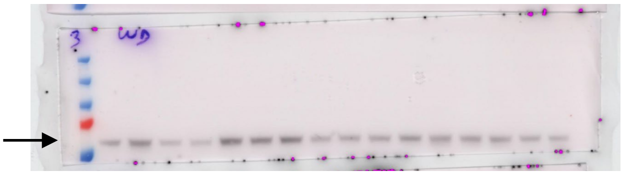

**LIVER LC3B**

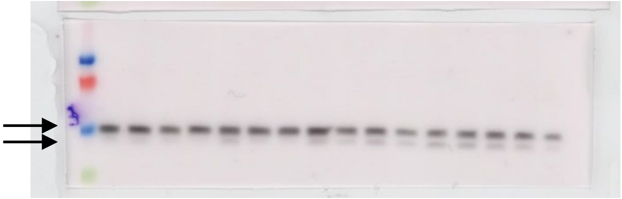

**LIVER GAPDH**

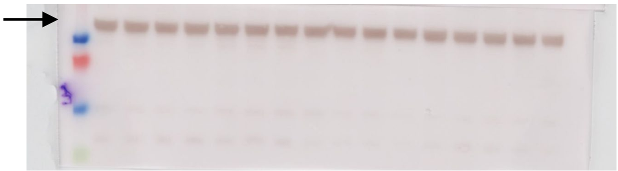

# Figure 5

**LIVER Col1a1**

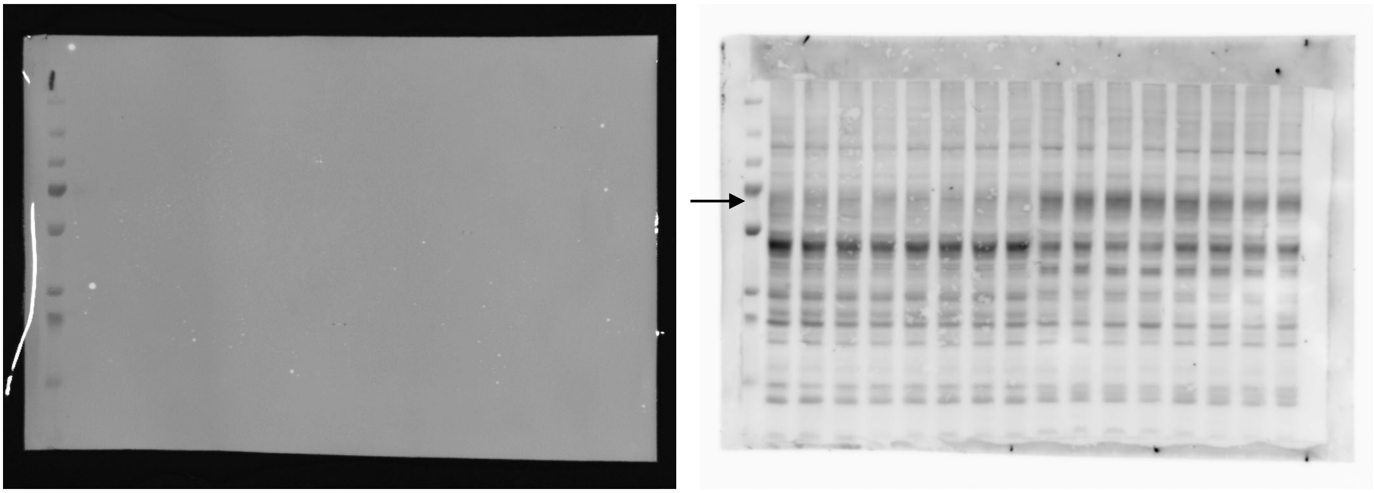

**LIVER aSMA**

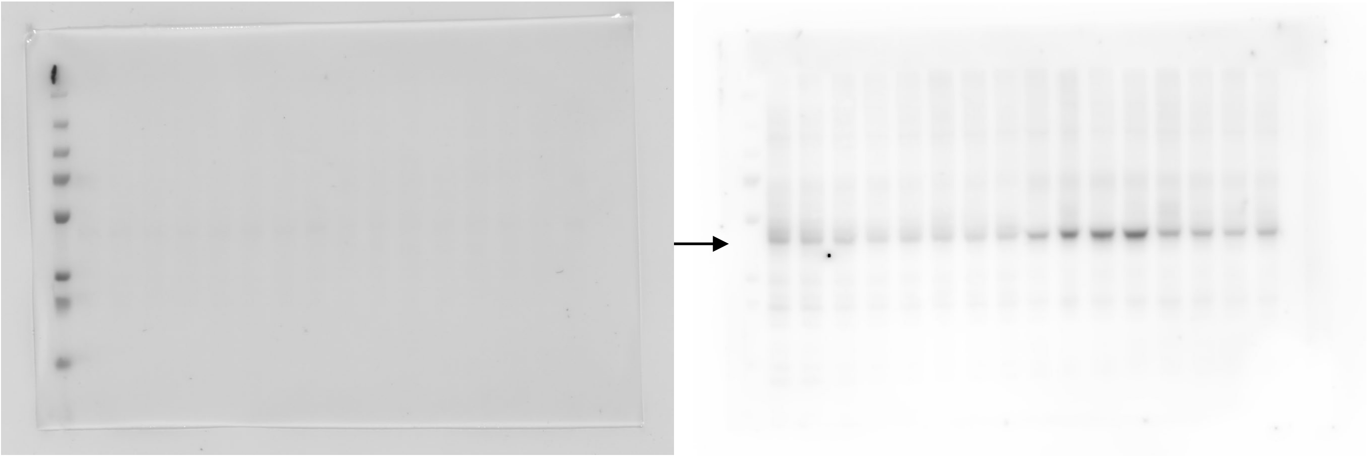

**LIVER GAPDH**

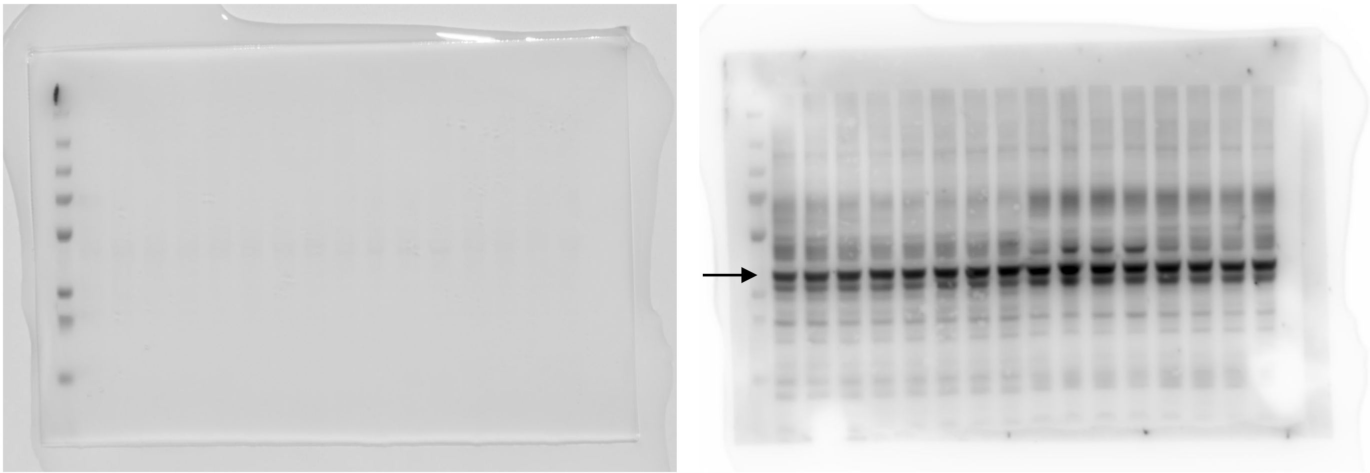

## Figure S3

LIVER p62

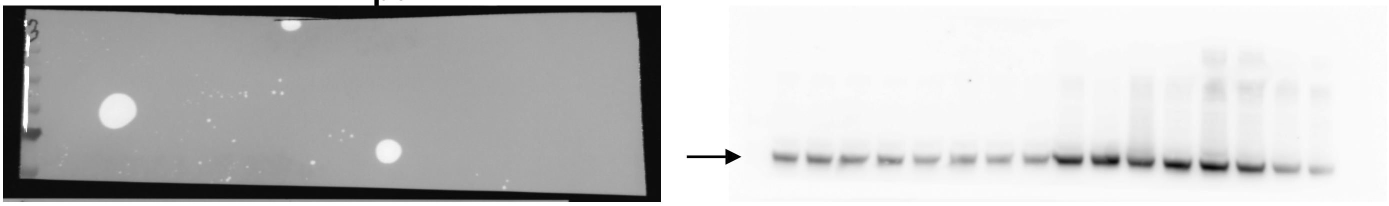

LIVER LC3B

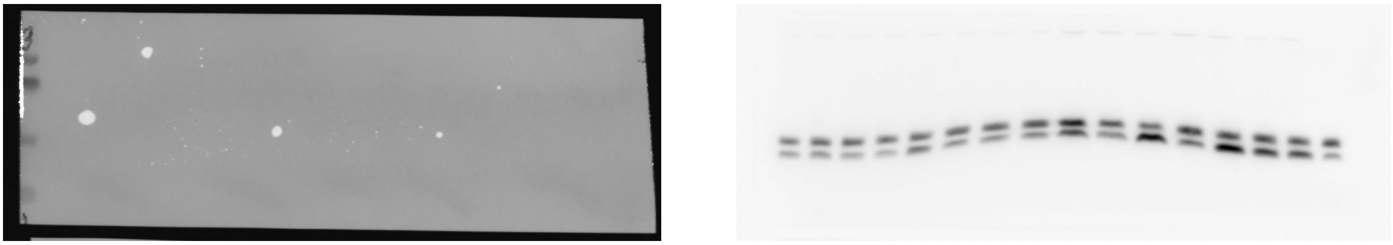

LIVER GAPDH

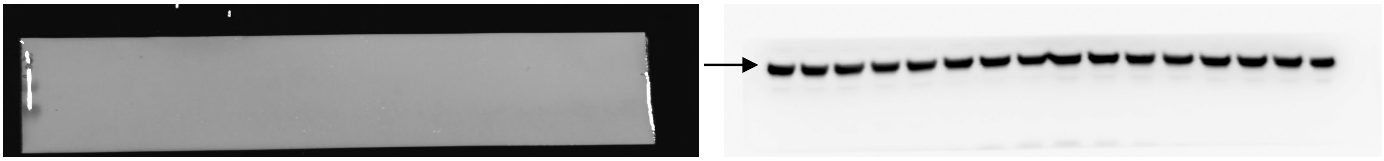

Supplement: Supplementary file 3 — Raw_WB [file 41418_2024_1410_MOESM3_ESM.pdf]
